# Genome-Wide Correlation of 36 Agronomic Traits in the 287 Pepper (*Capsicum*) Accessions Obtained from the SLAF-seq-Based GWAS

**DOI:** 10.3390/ijms20225675

**Published:** 2019-11-13

**Authors:** Lang Wu, Peng Wang, Yihao Wang, Qing Cheng, Qiaohua Lu, Jinqiu Liu, Ting Li, Yixin Ai, Wencai Yang, Liang Sun, Huolin Shen

**Affiliations:** 1 Beijing Key Laboratory of Growth and Developmental Regulation for Protected Vegetable Crops, China Agricultural University, Beijing 100193, China; 2 Department of Vegetable Science, College of Horticulture, China Agricultural University, No. 2 Yuanmingyuan Xi Lu, Haidian District, Beijing 100193, China

**Keywords:** pepper, agronomic traits, genome-wide association study (GWAS), specific-locus amplified fragment sequencing (SLAF-seq), multi-trait pyramid breeding, restorer-of-fertility (*Rf*) gene

## Abstract

There are many agronomic traits of pepper (*Capsicum* L.) with abundant phenotypes that can benefit pepper growth. Using specific-locus amplified fragment sequencing (SLAF-seq), a genome-wide association study (GWAS) of 36 agronomic traits was carried out for 287 representative pepper accessions. To ensure the accuracy and reliability of the GWAS results, we analyzed the genetic diversity, distribution of labels (SLAF tags and single nucleotide polymorphisms (SNPs)) and population differentiation and determined the optimal statistical model. In our study, 1487 SNPs were highly significantly associated with 26 agronomic traits, and 2126 candidate genes were detected in the 100-kb region up- and down-stream near these SNPs. Furthermore, 13 major association peaks were identified for 11 key agronomic traits. Then we examined the correlations among the 36 agronomic traits and analyzed SNP distribution and found 37 SNP polymerization regions (total size: 264.69 Mbp) that could be selected areas in pepper breeding. We found that the stronger the correlation between the two traits, the greater the possibility of them being in more than one polymerization region, suggesting that they may be linked or that one pleiotropic gene controls them. These results provide a theoretical foundation for future multi-trait pyramid breeding of pepper. Finally, we found that the GWAS signals were highly consistent with those from the nuclear restorer-of-fertility (*Rf*) gene for cytoplasmic male sterility (CMS), verifying their reliability. We further identified *Capana06g002967* and *Capana06g002969* as *Rf* candidate genes by functional annotation and expression analysis, which provided a reference for the study of cytoplasmic male sterility in *Capsicum*.

## 1. Introduction

The agronomic traits of pepper (*Capsicum* L.) have many categories with abundant phenotypes, which affect the roots, stem, leaves, flowers, and fruits [1,2,3]. Agronomic traits of pepper are closely related to pepper growth, and previous correlation and path analyses have indicated that fruit color, shape, and weight can be directly used as economic indicators and consumer purchase criteria for pepper products [4]. Plant type (Pt), branching habit (Bh), leaf shape (Ls), leaf color (Lc), first flower node (Ffn), number of flowers per axil (Nfpa), flowering date, and percentage of fertile fruit also affected the yield of pepper [5,6,7]. Stem and leaf pubescence (Lp) and dry matter, capsaicinoid, vitamin C, and anthocyanin content were correlated with resistance to disease and insect pests of pepper, which affected pepper production [8,9,10,11,12]. Therefore, mapping the genes that control agronomic traits is important for the development of the pepper industry (e.g., high-quality breeding and multi-trait pyramid breeding). However, most agronomic traits of pepper are controlled by quantitative trait loci (QTLs) and affected by environmental conditions, which makes it difficult to study these genes.

Previously, QTL mapping of agronomic traits in pepper has mainly focused on the agronomic traits closely associated with increasing pepper yield, such as fruit-related traits, disease resistance, and stress resistance (Table S1). For instance, using restriction fragment length polymorphism (RFLP), amplified fragment length polymorphism (AFLP), and random amplified polymorphic DNA (RAPD) markers, Ben Chaim et al., (2001) detected *fs3.1* controlling fruit shape in an F_3_ population [13]. Blum et al. (2003) detected *cap* controlling capsaicin content on chromosome 7 in an F_2_ population using RAPD and sequence characterized amplified regions (SCARs) markers [14]. There have also been several reports on QTL analyses of other agronomic traits, such as Pt, Bh, Ls, leaf margin (Lm), Ffn, and Nfpa [15,16,17]. However, there were no major QTLs detected, and the genes controlling QTLs have not been characterized.

In addition, some QTLs showed a cluster distribution in rice and in pepper [17,18], suggesting that these QTLs may be controlled either by one pleotropic gene or by a group of closely linked genes. Peterson (1959) found that the *A* gene (controlling purple color) was linked to the *sw* gene (controlling green color), and that both of them were linked to the *O* gene (controlling oblate fruit shape) in pepper [19]. Ben Chaim et al. (2003) mapped two anthocyanin loci (*Fc* and *A*) linked to a major quantitative trait locus, *fs 10.1*, for fruit-shape index [20]. These studies indicated that it is necessary to analyze the distribution of genes controlling the various traits of pepper, and then to explore the relationship between the traits at the genetic level.

The traditional QTL analysis, a gene mapping method, constructs separate populations (e.g., BC_1_, F_2_, F_3_, recombinant inbred line, and double haploid) and then maps the genes based on mass molecular markers (e.g., RFLP, RAPD, AFLP, simple sequence repeat (SSR), cleaved amplified polymorphic sequence (CAPS), and insertion/deletion (INDEL)) (Table S1). Therefore, it is labor intensive, time consuming, and expensive, and the location accuracy is limited by the genetic diversity of the parents and the density of molecular markers used in the different populations [21,22,23]. With the rapid development of high-throughput sequencing technology, genome-wide association study (GWAS) based on linkage disequilibrium has become another powerful tool for analyzing complex agronomic traits [23]. This approach can overcome the limitations of QTL analysis and finds the association between single nucleotide polymorphisms (SNPs) and phenotype by detecting gene (locus) imbalances in natural populations [24]. This method has been successfully applied to the identification of candidate genes controlling complex agronomic traits in plant species, such as wheat, rice, maize, soya bean, chickpea, tomato, cabbage, cucumber, and alfalfa [25,26,27,28,29,30,31,32]. There have been few reports on the application of GWAS in pepper, and most experiments focused on only one or several agronomic traits. Through analyzing 96 pepper accessions by GWAS, Nimmakayala et al. (2014) found one marker on chromosome 1, which was significantly associated with capsaicin and dihydrocapsaicin content [33]. Two years later, Nimmakayala et al. (2016a, 2016b) examined the weight per fruit (Wpf) and fruit pedicel length (Fpl) by GWAS. Their results showed that there were 16 and 36 SNPs significantly associated with the Wpf and Fpl, respectively [34,35]. Ahn et al. (2018) also mapped the genes associated with powdery mildew resistance on chromosome 4 by GWAS [36].

GWAS can result in a high frequency of false positive errors for screening SNPs associated with traits [37]. Therefore, some researchers have combined traditional QTL mapping with GWAS to study the traits of arabidopsis, rice, maize, winter faba bean, and soybean [28,38,39,40,41]. Such combined approaches successfully avoid the limitations of applying the two methods separately and increase the reliability of the results. This combined strategy has also been successfully applied to pepper, with Han et al. (2018) identifying five candidate genes involved in capsaicinoid biosynthesis in the regions found by GWAS and QTL analysis [42]. However, the measures taken by these studies to improve the reliability of GWAS results were based on only one aspect (validation methods) and did not consider material population or statistical models.

For species with larger genomes, GWAS is also expensive. Specific-locus amplified fragment sequencing (SLAF-seq) is a strategy for discovering SNPs facilitated by reduced-representation genome sequencing and next-generation sequencing technologies. SLAF-seq is not only cheaper, but also obtains markers with high coverage and can be typed among populations, providing an efficient method for the detection of SNP markers in species with larger genomes, such as wheat, hulless barley, sunflower, and pepper. Three agronomic traits of pepper have been analyzed by SLAF-seq: Ffn, resistance to *Phytophthora* root rot, and resistance to *Cucumber mosaic virus* [43,44,45]. These studies demonstrated the efficiency of SLAF-seq as a strategy for the identification of SNPs (or genes) in pepper.

In our study, 36 agronomic traits were investigated in 287 pepper accessions in a GWAS (Table S2 and Note S1). The rates of false positive errors in GWAS analyses can be high owing to population structure and relatedness [22]. Therefore, the following analyses were carried out successively: (1) genetic diversity analysis to evaluate the phenotypic and genetic variation; (2) distribution analysis for SLAF tags and SNPs to assess the quality of development markers; (3) determination of optimal statistical model for each agronomic trait; (4) large-scale GWAS for the 36 agronomic traits; (5) construction of physical map based on the SNPs significantly associated with the 36 agronomic traits to explore correlations among agronomic traits; (6) verification of GWAS results based on fine mapping of male-sterility (*Rf* gene) traits. Our objective was to identify candidate genes controlling agronomic traits important for pepper growing and explore the correlations among the 36 traits. Our findings provide key information for breeding high-quality pepper varieties and will be useful for developing multi-trait pyramid breeding strategies and increasing pepper yield.

## 2. Results

### 2.1. Genetic Diversity Analysis for the GWAS Population

In our study, 36 agronomic traits of pepper were surveyed and divided into 23 qualitative and 13 quantitative traits (Tables S3 and S4; Figures S1–S4). For the qualitative traits, 71 (80 in pepper germplasm resources) phenotypic types were identified, accounting for 88.75% of pepper germplasm resources (Table S3). The frequency distribution showed the main phenotype in those accessions (Figure S5). The coefficient of variation (CV) of 23 qualitative traits ranged from 0.09 to 0.74. Pt and Bh had the lowest CV (0.09), and fruit shoulder shape (Fss) had the highest CV (0.74). The Shannon Wiener index (H′) of 23 qualitative traits ranged from 0.14 (corolla color, Cc) to 1.24 (fruit surface furrow, Fsf). For the 13 quantitative traits, the frequency of Ffn, Fpl, and fruit length (Fl) were normally distributed (Figure S6). However, the frequency distribution of the other quantitative traits was positively skewed, indicating that a large proportion of the 287 pepper accessions had a lower value for these traits (Figure S6). In addition, mode, mean, min, max, range, CV, and H′ were used to assess the variation in quantitative traits (Table S4). The CV of 13 quantitative traits ranged from 0.26 (Fpl) to 1.07 (Wpf). The H′ of 13 quantitative traits ranged from 0.45 (Nfpa) to 2.01 (Fl). Our results confirmed that most of the 36 agronomic traits had a rich phenotypic variation, suggesting that this population met the requirements for GWAS analysis.

### 2.2. Identification and Distribution Analysis for Labels

In total, 2238.81 M paired-end reads were obtained from 287 pepper accessions based on HaeIII digestion. The average value of Q_30_ (Q_30_ indicates a quality score of 30, indicating a 0.1% error rate or 99.9% sequence accuracy) and GC content were 95.72% (93.62–96.6%) and 39.30% (38.51–42.42%), respectively, indicating that our sequencing results for 287 pepper accessions were reliable (Table S5). Further analysis revealed that 1,824,874 SLAF tags were obtained, with 287,910–475,273 tags for each accession. The average sequencing depth of each accession was different (12.17–44.22; average, 19.62) but met the assumptions of the SLAF test (Table S6). Furthermore, 1,025,395 polymorphic SLAF tags were identified from 1,824,874 SLAF tags, and then a total of 9,557,790 SNPs were also developed from polymorphic SLAF tags (see Table S7 for SNP details).

To analyze the distribution of genetic information of SLAF tags and SNPs in the 287 accessions, we divided all accessions into 17 grades according to SLAF tag number, SNP number, SNP integrity, and SNP heterozygosity ratio (Table S8). In the population, both SLAF tags and SNPs were normally distributed (Figure S7). The eighth SLAF tag grade (SLAF tags ranged from 365,060 to 376,081) had the highest frequency (95) and percentage (33.10%). The ninth SNP grade (SNPs ranged from 3,859,505 to 3,928,957) had the highest frequency (55) and percentage (19.16%). SNP integrity was also normally distributed. These results showed that SLAF tags and SNPs were evenly distributed in the population, implying that their genetic information covered most of the accessions. However, the distribution of SNP heterozygosity ratio was positively skewed in the population, indicating that most of the accessions had a lower heterozygosity ratio.

**Figure 1:**
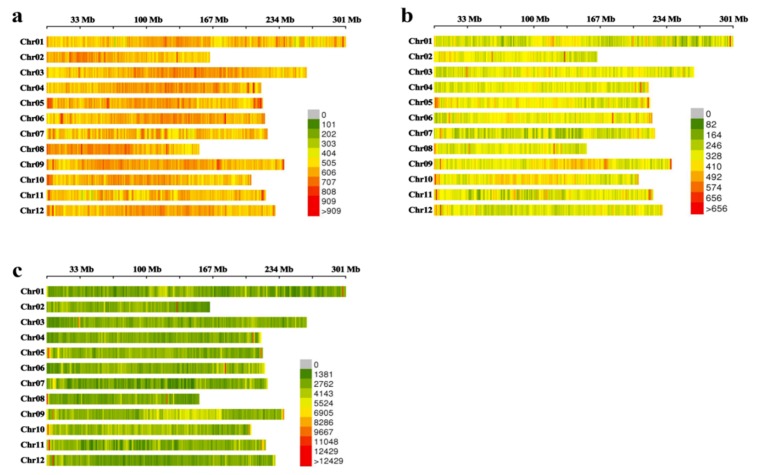
Density thermal map of specific-locus amplified fragment (SLAF), polymorphic SLAF (ploy-SLAF) and single nucleotide polymorphisms (SNPs) on chromosomes. (**a**) The number of SLAF within 1 Mb window size. (**b**) The number of poly-SLAF within 1 Mb window size. (**c**) The number of SNPs within 1 Mb window size. Color index indicates the number of labels.

As shown in Figure S8 and Figure 1, the distribution of SLAF tags and SNPs on chromosomes were also analyzed in our study. The percentage of polymorphic SLAF tags on every chromosome was over 50% of the total number of SLAF tags, and the percentage on chromosomes 5, 9, and 10 was high at 58.63%, 60.48%, and 63.46%, respectively. Chromosome 8 had the lowest percentage of polymorphic SLAF tags (53.13%) (Figure S8). The density thermal map of SLAF tags and polymorphic SLAF tags had fewer color types and balanced distribution (Figure 1). Similar results were observed in the density heat map of SNPs, with only a small number of regions exhibiting high density. These results suggested that all SLAF tags, polymorphic SLAF tags, and SNPs were evenly distributed on every chromosome. This reduced the probability of missing important information on different chromosomes, suggesting that theses labels can represent the genetic information of the whole pepper genome and be used for GWAS analysis of the agronomic traits of pepper.

### 2.3. Population Structure Analysis for the GWAS Population

In the present study, linkage disequilibrium was calculated for every chromosome using 594,429 SNPs (integrity >0.5; minor allele frequency (MAF) > 0.05). Squared correlations of allele frequencies (R^2^) were used to investigate the extent of LD calculated within a 0–500-kb window. As shown in Figure S9, the LD decay distance for the 287 pepper accessions between all SNPs was >500 kb when the value of the cut-off for R^2^ was set at 0.1, which showed that there was a greater probability of linkage among those SNPs in the accessions. In addition, the LD decays were not evenly distributed among chromosomes within the 0–500-kb window. However, obvious LD differentiation was found among chromosome 1 and the other chromosomes.

**Figure 2:**
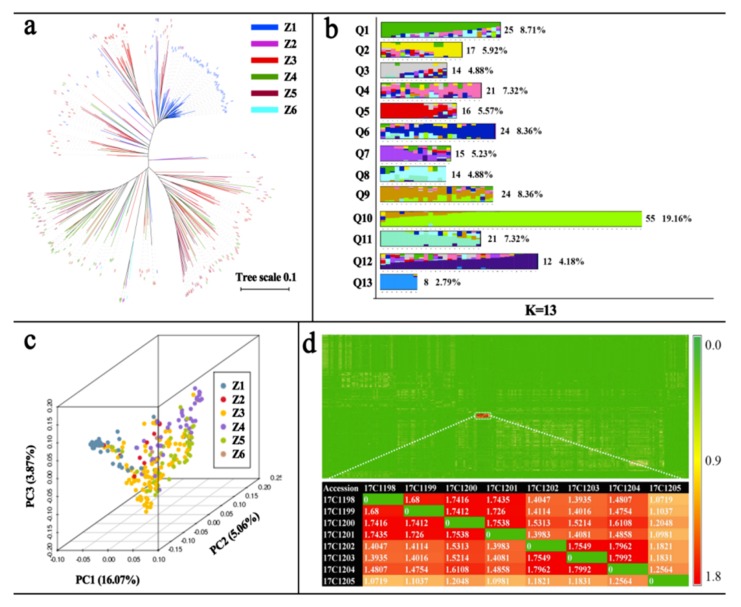
Population structure analysis for 287 pepper accessions. (**a**) NJ tree of 287 pepper accessions constructed from simple matching distance of genome SNPs. The Z1, Z2, Z3, Z4, Z5, and Z6 represent fruit shape is lantern, cons, horns (cattle and sheep horns), fingers, linear and the other, respectively. (**b**) Plots of the first three principal components of 287 pepper accessions. The Z1, Z2, Z3, Z4, Z5, and Z6 represent fruit shape is lantern, cons, horns (cattle and sheep horns), fingers, linear and the other, respectively. (**c**) Admixture in sub-populations (*K* = 13) resolved using 9,557,790 SNPs. The accessions were divided into 13 subgroups: Q1–Q13. The values represent accession number and percentage for each group. (**d**) Relatedness value thermal map of 287 pepper accessions. The color bar represents relatedness value.

Population structure and relatedness were the major factors leading to high rates of false positive errors in GWAS analysis [22]. Therefore, three methods were used to assess the population. First, a phylogenetic tree was constructed. As shown in Figure 2a, 287 pepper accessions were divided into four large categories and seven subcategories. Second, principal component analysis (PCA) was carried out. The interpretation rates of the first three principal components were 16.07% (PC1), 5.06% (PC2), and 3.87% (PC3), and cumulative variation was 25.01% (Figure S10a). The PCA triplot showed that most accessions were scattered except for one subgroup (Figure 2b, Gif S1). Third, admixture software was used to calculate the clustering from K = 1–15. As shown in Figure S10b, as the *K* value increased, the cross validation error rate rapidly decreased, the first valley value appeared at *K* = 11, the second valley value occurred at *K* = 13, and then the cross validation error rate increased gradually as *K* value increased. By comparing the value of cross validation error rate between *K* = 11 and *K* = 13, it was determined that *K* = 13, which had a lower cross validation error rate, was the optimal subgroup number for this population. Detailed information of the subgroups for *K* = 13 is shown in Figure 2c and Table S9. These results indicated a moderate level of population differentiation within different pepper subgroups.

As shown in Figure S10c, the relatedness of most accessions (76.44%) was <0.1 and was only >0.4 for 1.39% of accessions. The thermogram of relatedness also showed similar results with only eight accessions having a relatedness of >1.05 (Figure 2d). These results showed that most of the accessions were only distantly related, and thus, relatedness barely interfered with the GWAS analysis.

### 2.4. Large-Scale GWAS for 36 Agronomic Traits

The highly consistent filtered SNPs (594,429) in the 287 pepper accessions were used for GWAS analysis of 36 agronomic traits. To reduce the influence of population structure and increase the reliability of GWAS results, we used five statistical models: general linear model (GLM), mixed linear model (MLM), compressed mixed linear model (CMLM), efficient mixed-model association expedited (EMMAX), and factored spectrally transformed linear mixed model (FaST-LMM). Performance of the five models was compared based on Q-Q plots (Figures S11–S14), and the most appropriate statistical model for each trait was selected for subsequent GWAS analysis. As shown in Table 1, Fast-LMM was the optimal model of 20 traits; EMMAX was the optimal model of 11 traits; GLM was the optimal model of five traits. These results showed the optimal model of different traits can be different, implying that it was necessary for each trait to select one optimal model for GWAS analysis.

In total, 1487 SNPs were identified with *p* < 1.707 × 10^−8^ as the highly significant threshold from the optimal models, and 2126 candidate genes in the 100-kb region up- or down-stream near those SNPs were detected for 26 traits (Table S10) [46,47]. The number of highly significant associated SNPs and genes for each trait and chromosome varied (Table S11). In addition, 247 associated peaks (SNPs were arranged neatly with a columnar distribution, Figures S15–S20) were identified for the 36 traits, and the 109 associated peaks above the highly significant threshold (blue lines in Manhattan plots) were defined as significant peaks (strong association signals) for 26 traits. According to the number and physical location of SNPs in the significant peaks, 91 significant peak regions were identified for 22 traits (Table 1). The important SNPs and genes in the significant peak regions were highlighted in red (Table S10), and the important genes were annotated (Table S12).

#### 2.4.1. The Three Stem-Related Traits

##### Plant Type

**Figure 3:**
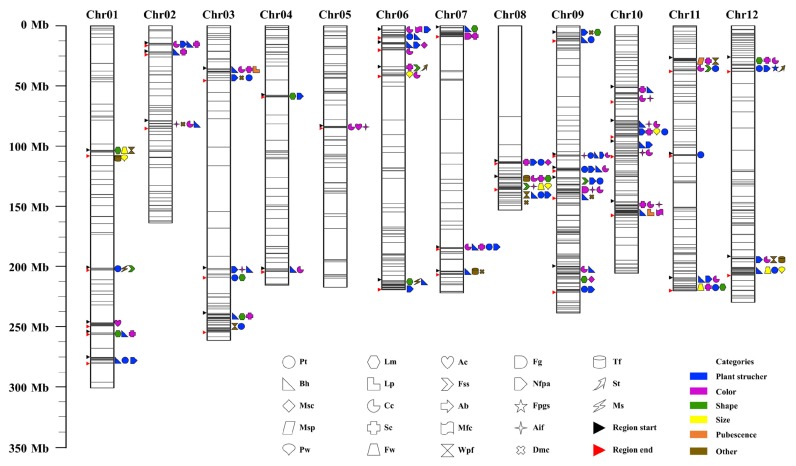
The position of 37 polymerization region for 23 agronomic traits on each chromosome. On the left aside of the chromosomes, the starting position of polymerization region was indicated by the black triangle, and the end position of that was indicated by the red triangle. Different colors and shapes represent the trait in various categories.

In total, 104 SNPs (184 genes) mainly distributed on chromosomes 1–9, 11, and 12 were significantly associated with Pt (Table S11). Chr11_37287718, Chr04_214680400, and Chr09_169528955 were the most highly associated with Pt (Table S10). Nineteen SNP polymerization regions were highlighted on chromosomes 1, 3, 6, 7, 8, 9, 11, and 12, in which the SNP associated with Pt and the SNP associated with the other traits had polymerization (Figure 3). Specifically, Pt and Bh, Pt and Cc, and Pt and Nfpa had 15, 16, and 13 polymerization regions, respectively (Table S17). Moreover, 10 highly significant peaks associated with Pt were observed on chromosomes 1, 5–7, 9, and 11, with no main peak evident (Figure S15). Furthermore, nine significant peak regions (total size: 17.19 Mbp), distributed on chromosomes 1, 4, 5, 6, 9, and 11 were identified for Pt (Table 1).

##### Branching Habit

In total, 307 SNPs (548 genes) distributed on all chromosomes were significantly associated with Bh (Table S11). Chr10_51277133, Chr03_240589834, and Chr10_99038533 were the most highly associated with Bh (Table S10). Then, 27 SNP polymerization regions were highlighted on chromosomes 1–4 and 6–12, in which the SNP associated with Bh and the SNP associated with the other traits had polymerization (Figure 3). Specifically, Bh and Lm, Bh and Cc, Bh and style color (Sc), Bh and Nfpa, and Bh and Aif had 9, 18, 12, 18, and 9 polymerization regions, respectively (Table S17). Moreover, eight highly significant peaks associated with Bh were observed on chromosomes 1–3, 6, 7, and 9, with no main peak evident (Figure S15). Furthermore, eight significant peak regions (total size: 20.92 Mbp), distributed on chromosomes 1, 2, 3, 6, 7, and 9, were identified for Bh (Table 1).

##### Main Stem Pubescence

In total, 22 SNPs (two genes) mainly distributed on chromosome 11 were significantly associated with main stem pubescence (Msp) (Table S11). Chr11_27217603, Chr11_27217695, and Chr11_27217636 were the most highly associated with Msp (Table S10). One SNP polymerization region was highlighted on chromosome 11, in which the SNP associated with Msp and the SNP associated with the other traits (Cc, Sc, Fss, and Wpf) had polymerization (Figure 3). Moreover, one highly significant peak on chromosome 11 was the main peak associated with Msp (Figure S19). Furthermore, one significant peak region (size: 1.57 Mbp), located on chromosome 11, was identified for Msp (Table 1).

#### 2.4.2. The Two Leaf-Related Traits

##### Leaf Margin

In total, 117 SNPs (247 genes) mainly distributed on chromosomes 1 and 3–12 were significantly associated with Lm (Table S11). Chr09_213296577, Chr08_134733299, and Chr10_59043555 were the most highly associated with Lm (Table S10). Twelve SNP polymerization regions were highlighted on chromosomes 1, 3, 4, 6, 7, 8, 9, 11, and 12, in which the SNP associated with Lm and the SNP associated with the other traits had polymerization (Figure 3). Specifically, Lm and Cc, Lm and Sc, and Lm and Nfpa had 4, 6, and 8 polymerization regions, respectively (Table S17). Moreover, five highly significant peaks were observed on chromosomes 1, 4, 6, 8, and 12, with no main peak evident (Figure S17). Furthermore, five significant peak regions (total size: 11.18 Mbp), distributed on chromosomes 1, 4, 6, 8, and 12 were identified for Lm (Table 1).

##### Leaf Pubescence

In total, 17 SNPs (51 genes) mainly distributed on chromosomes 2, 3, 9, 10, and 12 were significantly associated with Lp (Table S11). Chr10_154508628, Chr09_178710956, and Chr10_197927729 were the most highly associated with Lp (Table S10). Two SNP polymerization regions were highlighted on chromosomes 3 and 10, in which the SNP associated with Lp and the SNP associated with the other traits had polymerization (Figure 3). Specifically, Lp and Cc, and Lp and Sc had two polymerization regions, respectively (Table S17). Moreover, three highly significant peaks were observed on chromosomes 10 and 12, with the former being the main peak associated with Lp (Figure S19). Furthermore, three significant peak regions (total size: 3.00 Mbp), distributed on chromosomes 10 and 12, were identified for Lp (Table 1).

#### 2.4.3. The Five Flower-Related Traits

##### Corolla Color

In total, 125 SNPs (279 genes) distributed on all of chromosomes were significantly associated with Cc (Table S11). Chr02_85429287, Chr09_201616976, and Chr08_138467669 were the most highly associated with Cc (Table S10). Then, 22 SNP polymerization regions were highlighted on chromosomes 2–12, in which the SNP associated with Cc and the SNP associated with the other traits had polymerization (Figure 3). Specifically, Cc and Sc, Cc and Nfpa, and Cc and Aif had 11, 15, and 9 polymerization regions, respectively (Table S17). Moreover, eight highly significant peaks were observed on chromosomes 2, 4, 6–9, and 11, with no main peak evident (Figure S16). Furthermore, nine significant peak regions (total size: 8.18 Mbp), distributed on chromosomes 2, 4, 6, 7, 8, 9 and 11, were identified for Cc (Table 1).

##### Style Color

In total, 291 SNPs (152 genes) distributed on all of chromosomes were significantly associated with Sc (Table S11). Chr10_157424507, Chr10_155327941, and Chr10_155327958 were the most highly associated with Sc (Table S10). Sixteen SNP polymerization regions were highlighted on chromosomes 1, 2, 3, 6, 7, 8, 10, 11, and 12, in which the SNP associated with Sc and the SNP associated with the other traits had polymerization (Figure 3). Specifically, Sc and Nfpa, Sc and Aif had 8 and 4 polymerization regions, respectively (Table S17). Moreover, six highly significant peaks were observed on chromosomes 1–3, 8, 10, and 11, with the latter two being the main peaks associated with Sc (Figure S16). Furthermore, three significant peak regions (total size: 1.81 Mbp), distributed on chromosomes 1, 10, and 11, were identified for Sc (Table 1).

##### Number of Flowers Per Axil

In total, 126 SNPs (221 genes) distributed on all of chromosomes were significantly associated with Nfpa (Table S11). Chr12_193629045, Chr12_193629029, and Chr12_193628684 were the most highly associated with Nfpa (Table S10). Then, 21 SNP polymerization regions were highlighted on chromosomes 1–4 and 6–12, in which the SNP associated with Nfpa and the SNP associated with the other traits had polymerization (Figure 3). Specifically, Nfpa and Aif, Nfpa and dry matter content (Dmc) had 6 and 4 polymerization regions, respectively (Table S17). Moreover, 11 highly significant peaks were observed on chromosomes 1, 4–7, 9–10, and 12, with the latter being the main peak associated with Nfpa (Figure S15). Furthermore, 12 significant peak regions (total size: 18.48 Mbp), distributed on chromosomes 1, 4, 5, 6, 7, 9, 10, 11, and 12, were identified for Nfpa (Table 1).

##### Flower Pedicel Growing State

In total, 16 SNPs (14 genes) distributed on chromosome 12 were significantly associated with flower pedicel growing state (Fpgs) (Table S11). Chr12_37932067, Chr12_37994286, and Chr12_37994288 were the most highly associated with Fpgs (Table S10). One SNP polymerization region was highlighted on chromosome 12, in which the SNP associated with Fpgs and the SNP associated with the other traits (Lm, Sc, Cc, Pt, Nfpa, and spicy type (St)) had polymerization (Figure 3). Moreover, one highly significant peak on chromosome 12 was the main peak associated with Fpgs (Figure S15). Furthermore, one significant peak region (size: 5.14 Mbp), located on chromosome 12, was identified for Fpgs (Table 1).

##### Male Sterility

In total, 78 SNPs (17 genes) mainly distributed on chromosomes 1, 6, 10, and 11 were significantly associated with male sterility (Ms) (Table S11). Chr06_215261283, Chr06_215468191, and Chr06_215411595 were the most highly associated with Ms (Table S10). Two SNP polymerization regions were highlighted on chromosomes 1 and 6, in which the SNP associated with Ms and the SNP associated with the other traits had polymerization (Figure 3). Moreover, one highly significant peak on chromosome 6 was the main peak associated with Ms (Figure S20). Furthermore, one significant peak region (size: 1.09 Mbp), located on chromosome 6, was identified for Ms (Table 1).

#### 2.4.4. The Five Fruit-Related Traits

##### Anthocyanin on Immature Fruit

In total, 102 SNPs (96 genes), mainly distributed on chromosomes 1–5 and 7–10, were significantly associated with anthocyanin on immature fruit (Aif) (Table S11). Chr07_105839253, Chr10_75362680, and Chr10_107762896 were the most highly associated with Aif (Table S10). Ten SNP polymerization regions were highlighted on chromosomes 2, 3, 5, 9, and 10, in which the SNP associated with Aif and the SNP associated with the other traits had polymerization (Figure 3). Specifically, Aif and Fss, Aif and placenta width (Pw), and Aif and Dmc had 2, 2, and 3 polymerization regions, respectively (Table S17). Moreover, seven highly significant peaks were observed on chromosomes 2, 4, and 7–10, with the latter being the main peak associated with Aif (Figure S16). Furthermore, four significant peak regions (total size: 7.59 Mbp), distributed on chromosomes 8, 9, and 10, were identified for Aif (Table 1).

##### Mature Fruit Color

In total, 16 SNPs (49 genes) distributed on chromosome 6 were significantly associated with mature fruit color (Mfc) (Table S11). Chr06_10060744, Chr06_8776355, and Chr06_8769243 were the most highly associated with Mfc (Table S10). Two SNP polymerization regions were highlighted on chromosomes 6 and 10, in which the SNP associated with Mfc and the SNP associated with the other traits had polymerization (Figure 3). Moreover, one highly significant peak on chromosome 6 was the main peak associated with Mfc (Figure S16). Furthermore, one significant peak region (size: 6.30 Mbp), located on chromosome 6, was identified for Mfc (Table 1).

##### Spicy Type

In total, 44 SNPs (29 genes) mainly distributed on chromosomes 3, 4, 9, 10, and 12 were significantly associated with St (Table S11). Chr12_35372851, Chr12_35623982, and Chr12_35624024 were the most highly associated with St (Table S10). Two SNP polymerization regions were highlighted on chromosomes 6 and 12, in which the SNP associated with St and the SNP associated with the other traits had polymerization (Figure 3). Moreover, six highly significant peaks were observed on chromosomes 3, 4, 9, 10, and 12, with the latter being the main peak associated with St (Figure S20). Furthermore, six significant peak regions (total size: 8.22 Mbp), distributed on chromosomes 4, 5, 6, 10, and 12, were identified for St (Table 1).

##### Placenta Width

In total, seven SNPs (18 genes) distributed on chromosomes 1, 6, 8, and 12 were significantly associated with Pw (Table S11). Chr12_206591342, Chr06_40911519, and Chr08_133396929 were the most highly associated with Pw (Table S10). Five SNP polymerization regions were highlighted on chromosomes 1, 6, 8, 10 and 12, in which the SNP associated with Pw and the SNP associated with the other traits had polymerization (Figure 3). Specifically, Pw and fruit width (Fw), Pw and Wpf, and Pw and thickness of flesh (Tf) each had three polymerization regions (Table S17). Moreover, four highly significant peaks were observed on chromosomes 1, 8, and 12, and two of them (on chromosomes 8 and 12) were the main peaks associated with Pw (Figure S18). Furthermore, two significant peak regions (total size: 3.27 Mbp), distributed on chromosomes 8 and 12, were identified for Pw (Table 1).

##### Fruit Width

In total, 26 SNPs (65 genes) distributed on chromosomes 1, 4, 8, and 12 were significantly associated with Fw(Table S11). Chr08_133396929, Chr08_133433188, and Chr08_133185108 were the most highly associated with Fw (Table S10). Four SNP polymerization regions were highlighted on chromosomes 1, 8, 11, and 12, in which the SNP associated with Fw and the SNP associated with the other traits had polymerization (Figure 3). Specifically, Fw and Wpf, and Fw and Tf each had three polymerization regions (Table S17). Moreover, four highly significant peaks were observed on chromosomes 1, 4, 8, and 12, with the peak on chromosome 8 being the main peak associated with Fw (Figure S18). Furthermore, three significant peak regions (total size: 6.68 Mbp), distributed on chromosomes 1, 8, and 12, were identified for Fw (Table 1).

There were few to no SNPs significantly associated with the remaining 21 traits: main stem color (Msc), Ls, Lc, leaf surface characteristics (Lsc), Ffn, anther color (Ac), fruit glossy (Fg), Fsf, fruit surface characteristics (Fsc), Fss, appendage at blossom-end (Ab), Fpl, placenta length (Pl), Fl, fruit apex shape (Fas), fruit shape index (Fsi), placenta size index (Psi), Wpf, Dmc, Tf, and number of locules (Nol). However, there were many peaks with relatively small effects observed distributed on multiple chromosomes (Figures S15–S18, S20). These findings implied that these traits may be controlled by multiple micro-genes.

### 2.5. Global Exploration of Correlations Among the 36 Agronomic Traits

**Figure 4:**
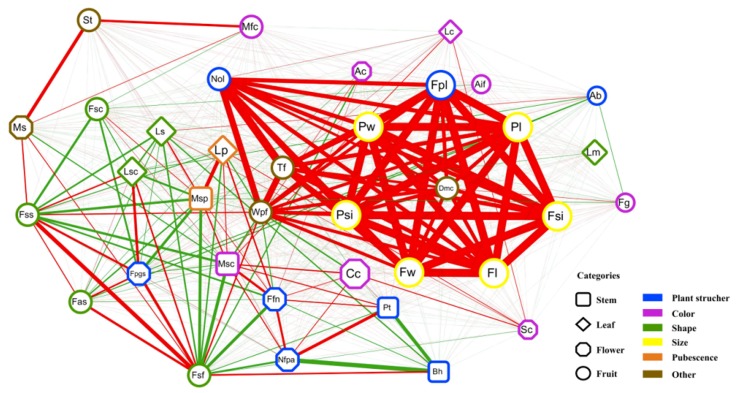
Phenotype correlation network of agronomic traits in pepper. Red and green lines represent negative and positive correlations, respectively. Line width is proportional to the strength of the correlation. Different shape nodes represent stem-related, leaf-related, flower-related and fruit-related traits, respectively. Different color frames of nodes represent plant structure-related, color-related, shape-related, size-related, pubescence-related and other traits, respectively.

We explored the correlations among the 36 traits using correlation network analysis (Figure 4). The strongest positive correlation was observed among the 11 fruit-related traits: Fpl, Pw, Pl, Psi, Fw, Fl, Fsi, Nol, Wpf, Tf, and Dmc. For stem-related traits, Bh was negatively correlated with Pt (−0.49), and Msc was positively correlated with Msp (0.25) and Pt (0.23). Correlations among leaf-related traits and flower-related traits were less significant than those among stem-related traits. Correlation coefficients among pairwise traits are shown in Table S14.

We compared their shared peak numbers in Manhattan plots based on descriptive classifications (Figures S15–S20). For plant structure-related traits, Pt, Bh, and Nfpa had a total of three shared peaks distributed on chromosomes 1, 6, and 7. However, there was one shared peak on chromosome 2 for Bh and Fpgs, and one shared peak on chromosome 12 for Fpl and Fpgs.

For color-related traits, Cc and Msc, and Cc and Fg each had one shared peak distributed on chromosomes 9 and 8, respectively. Cc, Lc, and Fg had one shared peak on chromosome 12. Cc, Ac, and Aif had one shared peak on chromosome 2. Sc and Msc had one shared peak on chromosomes 8 and 10, respectively. Sc, Aif, and Mfc had one shared peak on chromosome 10. Ac and Aif had one shared peak on chromosomes 4 and 7, respectively. Msc and Mfc had one shared peaks on chromosomes 10. 

For shape-related-traits, Ls and Lsc had one shared peak on chromosome 6. Ls and Fas had four shared peaks distributed on chromosomes 1, 2, 10, and 12. Moreover, Fsc and Fsf, and Fsc and Fas each had one shared peak on chromosomes 10 and 12, respectively. 

For organ size-related traits, Fw and Pw had four shared peaks on chromosomes 1, 4, 8, and 12. Fl and Pl had two shared peaks distributed on chromosomes 6 and 8. Fsi and Psi had one shared peak on chromosomes 2 and 7, respectively. 

For pubescence-related traits, Msp and Lp had one shared peak on chromosome 10, and it was the main peak of Lp. However, the main peak of Msp was on chromosome 11, suggesting that the major genes controlling the two traits may be different. There were more peaks for Lp (eight) than Msp (two), implying that Lp was controlled by minor genes. 

For other traits, there were six shared peaks between Wpf and Tf on chromosomes 1, 3, 5, 8, 11, and 12. St and Tf had one shared peak on chromosomes 3 and 8, respectively. St and Dmc, and St and Ms each had one shared peak each on chromosomes 6 and 2, respectively. We compared the distribution of peaks of the traits between the different categories and observed one or two shared peaks for Sc and Fsf (chromosome 10), Sc and Fss (chromosomes 8 and 11), Fas and Fg (chromosome 12), and St and Fpgs (chromosome 12). It was worth noting that Sc, Fss, and Msp had one shared peak (chromosome 11), that Sc, Aif, and Lp had one shared peak (chromosome 10), and that Sc, Cc, Fw, and Pw had one shared peak (chromosome 08).

**Figure 5:**
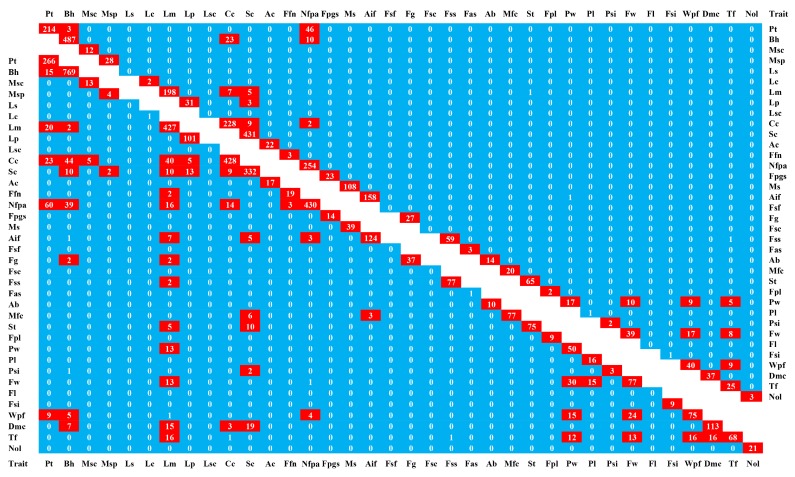
Significant SNPs and potential candidate gene numbers in pairwise traits. The upper triangle is the significant SNPs numbers in pairwise trits; The lower triangle is the potential candidate gene numbers in pairwise traits. The color bar represents the number of SNPs or candidate genes in pairwise traits.

Additionally, we identified the significant SNPs near peaks for pairwise traits and summarized the SNP and gene numbers. As shown in Figure 5, we found that among the significant SNPs, some of them were associated with more than one trait. For instance, 46 SNPs (60 genes) were associated with Pt and Nfpa, and 10 SNPs (39 genes) were associated with Bh and Nfpa. Based on pairwise traits, 385 genes were associated with more than one trait, indicating gene pleiotropy. For detailed information, see Table S15.

To further confirm the relationships among agronomic traits at the whole genome level, 2526 significant SNPs (*p* < 1.707 × 10^−7^) were mapped independently on chromosomes 1–12. We found that some of the SNPs clustered in a small region on a chromosome, which was deemed to be an SNP polymerization region. Totally, 37 SNP polymerization regions (total size: 264.69 Mbp) were found in the study, which could be selected areas in pepper breeding (Table S16). As shown in Figure 3, most SNP polymerization regions were distributed on chromosomes 1, 6, 9, and 10. The polymerization regions associated with more than four traits were mainly distributed on chromosomes 6–12 (Table S16). Compared with the correlation analysis results, we found that the stronger the correlation between two traits, the greater the possibility of them being in more than one polymerization region, and the higher the number of SNPs and genes in the same polymerization region (Figure 3, Figure 4 and Figure 5 and Table S17). For example, Bh and Nfpa were negatively correlated (−0.59), and there were 18 different shared regions for them. Similar features were also found between Pt and Nfpa (16 regions), and Pt and Bh (15 regions) (Table S17).

### 2.6. Verification of GWAS Results Based on Fine Mapping of Male-Sterility (Rf) Gene

**Figure 6:**
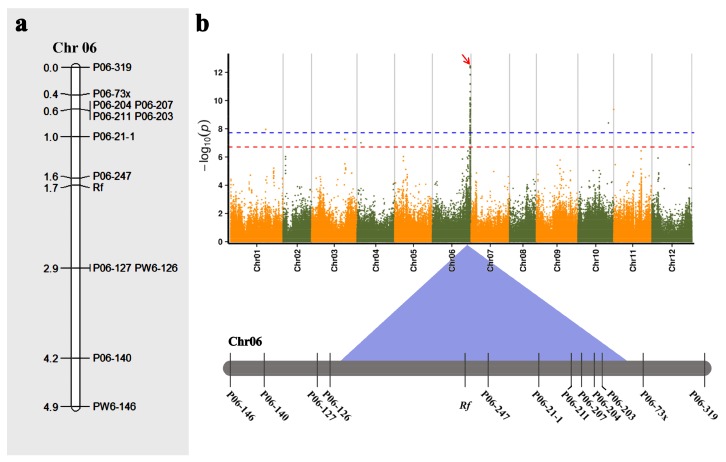
Comparison of molecular mapping with GWAS signals for Rf gene. (**a**) the molecular mapping result of Rf. (**b**) Comparison of the results of molecular mapping and GWAS mapping of Rf (factored spectrally transformed linear mixed (FaST-LMM) model). The red arrow represents the major peaks associated with male-sterility (Rf gene). The highly significant threshold is shown as a dash blue line (*p* < 1.707 × 10^−8^) and the significant threshold is shown as a dash red line (*p* < 1.707 × 10^−7^).

To verify the GWAS signals, we used molecular mapping of the *Rf* gene for cytoplasmic male sterility. In the F_2_ population of 2016, 255 individuals were fertile, and 79 were sterile. A chi-squared test revealed that the ratio of fertile:sterile individuals in this population was 3:1. The *Rf* gene was initially mapped within 4.9 cM between the SSR markers P06-319 and PW6-146 (Figure 6a), with a physical distance of 1.8 Mb. In the F_2_ population of 2017, the recombinant single strains were screened with P06-319 and PW6-146 as two side markers, and 116 individuals were recombinant between these markers. Genotypic assay of the recombinants was performed using 10 SSR markers between P06-319 and PW6-146. Finally, the *Rf* gene was mapped to a region between the markers P06-247 and PW6-126. This region lies within 214,868,888–215,727,145 bp of chromosome 6 in the ‘Zunla-1′ genome, with a physical distance of 858,257 bp. Comparing the regions of molecular mapping with the GWAS signals, we found that the region of molecular mapping (214,868,888–215,727,145) was highly consistent with that of GWAS (214,443,037–215,536,517) (Figure 6b), which indicated that the GWAS signals were reliable.

According to *Rf* gene localization, the 214,868,888–215,727,145-bp region of the ‘Zunla-1’ genome was annotated, and eight genes were found in this region (Table S13). cDNA of 138A and 138C were used as templates to detect whether there were differences in the coding sequences of the genes. The sequencing results showed that there were no differences in the coding sequences of the eight genes between the two templates.

The results of expression analysis showed that no expression was detected for *Capana06g002963* and *Capana06g002971*. *Capana06g002965* was expressed in both anthers and leaves, and its expression in anthers gradually decreased with bud development but showed no significant difference between 138A and 138C (Figure S21a). *Capana06g002967* was mainly expressed in buds, and the expression level gradually increased with bud development. The expression level of 138C was significantly higher than that of 138A at all stages of bud development (Figure S21b). *Capana06g002968* was mainly expressed in buds, and the highest expression was found in the anthers of 138A at the mature stage (Figure S21c). *Capana06g002969* was expressed in both anthers and leaves, and the anther expression in all stages of 138C was significantly higher than that in 138A (Figure S21d). *Capana06g002970* was expressed in both anthers and leaves, but there was no significant difference between 138A and 138C (Figure S21e). *Capana06g002972* was expressed in both anthers and leaves, and the highest expression was in the anthers of 138C at maturity and was significantly higher than that of 138A (Figure S21f). Based on the results, *Capana06g002967* and *Capana06g002969* were identified as *Rf* candidate genes.

## 3. Discussion

There are many agronomic traits with abundant phenotypes in pepper. However, most of the previous studies on the gene mapping of agronomic traits in pepper were based on the traditional QTL mapping method, and only a single or several traits were studied in the same study [48,49,50,51]. Although, there are also reports of the use of GWAS in pepper, most of this research was only focused on one or two traits [33,43,52]. In our study, the GWAS of 36 agronomic traits was carried out at the same time. A large number of SNP loci significantly associated with agronomic traits were obtained, and the main correlation peaks of some key agronomic traits were identified. Our results showed that GWAS could be used to study multiple agronomic traits in the same study, which greatly improved the efficiency of gene mapping of agronomic traits in pepper.

However, GWAS does have the potential for false positive errors due to the population structure, relatedness, and inappropriate statistical models [22,37,53,54]. From previous studies, we summarized three aspects that may contribute to improving the accuracy of GWAS results and that should be considered carefully, including population materials [25,30,55,56], statistical methods [22,57], and validation methods [43,52]. Most previous studies only considered verification methods, not material population or statistical models. To minimize the rates of false positives and false negatives and to ensure the reliability of the GWAS results, we carried out four measures based on those three aspects. We used molecular mapping of the *Rf* gene for cytoplasmic male sterility as an example to verify GWAS signals. Comparing the regions of molecular mapping with GWAS signals, we found that the region of molecular mapping was highly consistent with that of GWAS, indicating that the GWAS signals were reliable. Furthermore, using SSR molecular marker technology combined with the GWAS results of 287 pepper samples, the *Rf* gene was finally located in the region of 858,257 bp (214,868,888–215,727,145 bp) on chromosome 6 of the ‘Zunla-1′ genome, which was consistent with the two regions of 211.29–216.45 Mb and 215.11–215.68 Mb reported previously [58,59]. Additionally, in the simple comparison of the GWAS results of other traits with those of the traditional methods (Table S18), we found that more than one loci identified by the two methods (QTL and GWAS) for the same traits were located on the same chromosome, especially for some quantitative traits controlled by poly-genes, which also demonstrates the reliability of the GWAS result.

Pubescence is a key agronomic trait of pepper, which can improve drought resistance and protect plants from UV radiation, insects, and pathogenic bacteria [48,60]. Kim et al. (2010) found a major QTL (*Ptl1*) for stem pubescence on chromosome 10 [48]. Chunthawodtiporn et al. (2018) also found some QTL for pubescence on chromosomes 2, 10, and 11 and identified two candidate genes controlling pubescence formation on chromosome 10 [60]. In our study, pubescence was divided into Msp and Lp, and we detected correlation peaks for Msp on chromosomes 10 and 11, the latter being the major peak. However, the major peak associated with Lp was detected on chromosome 10. Those results showed that there was more than one major gene controlling the pubescence of pepper, and that genes may be specific to growth sites. 

Mfc is an important agronomic trait of pepper. Our results showed that the major peak for Mfc was on chromosome 6, where the *y* gene controls the red or yellow mature fruit [61]. However, the *c-2* gene controlling red or orange mature fruit was detected on chromosome 4 [61]. The *cl* gene, preventing chlorophyll degradation and controlling brown or olive-green mature fruit, was detected on chromosome 1 [62]. Aif is the stress response of pepper fruit to low temperature and strong UV radiation (Figure S4a), and its formation is mainly related to the accumulation of anthocyanin in fruit epidermis [63]. Genes regulating anthocyanin biosynthesis have been studied in many solanaceous plants, such as tomato, potato, eggplant, and pepper [64,65]. In pepper, a QTL for anthocyanin on immature fruit has been detected on chromosome 10, and a candidate gene was proposed for anthocyanin accumulation [60,66]. In this study, we found peaks of Aif on the same chromosome. Those results showed that the genetic and regulatory mechanism of fruit color is more complex, and there are differences among different mature fruit colors in pepper.

Most agronomic traits of pepper are controlled by QTLs with complex correlations among them, causing difficulties for breeding programs and hindering the development of multi-trait pyramid breeding. Previous studies found that the genes controlling the color of different parts of pepper plants were aggregated on chromosomes and linked to the genes controlling fruit shape [13,19,67]. Ben Chaim et al. (2001) also found that markers in two genomic regions linked to QTL for *Cucumber mosaic virus* resistance were also linked to QTL for fruit weight [13]. Our study analyzed the relationship among 36 agronomic traits of pepper from different angles. First, the correlation analysis based on phenotypic data showed that agronomic traits were highly correlated with each other, and the most significant correlations were observed for fruit-related traits, which is consistent with the results from previous research [68]. Correlation studies can help to determine the traits on which selection should be based to breed peppers for specific purposes [69]. Second, we compared the distribution of peaks in the Manhattan plots and found that there was more than one shared peak among different traits, suggesting that the traits were linked, and controlled either by one pleiotropic gene or by a group of closely-linked genes. Hence, we further analyzed the distribution of SNPs that were significantly associated with the 36 agronomic traits and were found in SNP polymerization regions on chromosomes, consistent with previous research results [17]. Furthermore, 37 SNP polymerization regions were found, which may be the main regions of natural selection and artificial selection in the long-term evolution of pepper. These SNP polymerization regions can be the focus of molecular marker development in the process of multi-trait pyramid breeding to improve the efficiency of multi-trait pyramid breeding.

In summary, we obtained many SNPs associated with agronomic traits and genes near the SNPs, which provided a large amount of biological information for the study of agronomic traits in pepper. Furthermore, we comprehensively explored the correlations among different agronomic traits and found 37 SNP polymerization regions, which laid a theoretical foundation for future multi-trait pyramid breeding of pepper. In future research, we will analyze the genetic effect of SNPs in these regions and further clarify the association among different traits and the pleiotropism of candidate genes.

## 4. Materials and Methods 

### 4.1. Global Exploration of Correlations among the 36 Agronomic Traits

In our study, we used 287 pepper accessions provided by the pepper research laboratory of the College of Horticulture, China Agricultural University for the global exploration of correlations among the 36 agronomic traits and the subsequent GWAS analysis. The 287 accessions were classified into *C. baccatum* (1), *C. chinense* (4), *C. frutescens* (4), and *C. annuum* (278). There were 13 F_1_ hybrids and 274 high-generation inbred lines (Table S2). All were planted three times with three replications in the greenhouses: once in 2017, 2018, and 2019. In each year, three replications (each replication with 12 plants) for each accession were grown in a randomized design in greenhouses and 3–6 plants were selected from each replication for surveying agronomic traits. The 36 agronomic traits were surveyed during different growing periods, details of the survey methods for agronomic traits are provided in Note S1 and Figures S1–S4. 

Analysis of correlations among 36 agronomic traits was performed by the R software (version 3.5.0, Ross Ihaka and Robert Gentleman, University of Auckland, Auckland, New Zealand) base on the phenotypic data of three years. As shown in Table S2, highly consistent qualitative trait indicator data and mean values of nine replications over three year quantitative trait indicator were used for correlation analysis, because there was no extremely significant difference (*p* < 0.01) in their phenotypic data over 3 years (Table S2). Furthermore, the correlation network procedure was carried out using the R package “qgraph” [70].

### 4.2. Analysis of Phenotypic Data

Phenotypic variation of the population was analyzed and evaluated using the frequency distribution and CV of the phenotypes of each trait. To evaluate the population diversity of the 287 pepper accessions, the Shannon-Weaver diversity index (H′) was calculated in Microsoft Excel 2007 version (MSO: 12.0.4518.1014) as follows:

(1) $$H^{\prime }=-\sum_{i=1}^{S}Pi\ln Pi$$

where *P_i_* is the percentage of materials contained in class *i* for a quantitative trait type indicator. For qualitative trait type indices, *Pi* is the percentage of materials, of which, phenotype was assigned a value equal to *i* (details of assignment standards are shown in Table S19). S is the number of grades for quantitative indicators, or the number of assigned values for qualitative trait type indicators.

Descriptive statistics (e.g., mode, mean, and max) were calculated and graphs drawn in Microsoft Excel 2007. Results are presented as the mean of nine replications over 3 years.

### 4.3. Extraction of Genomic DNA

Genomic DNA was isolated from six fresh leaves from plants using the cetyltrimethylammonium bromide method [71,72]. A NanoDrop 2000 spectrophotometer (Thermo Scientific, Waltham, MA, USA) was used to determine the DNA concentration and quality to ensure that DNA samples met the requirements of the sequencing reaction (concentration ≥ 20 ng/µL; volume ≥ 30 µL).

### 4.4. Design of Enzyme Digestion Scheme

The genome of ‘Zunla-1′ (http://peppersequence.genomics.cn/page/species/index.jsp, version 2.0) was selected as the reference genome for an electronic digestion prediction experiment. The genome size of ‘Zunla-1′ was about 3.36 Gb, and GC content was 34.97%. The principles for selection of the enzyme digestion scheme were as follows: (1) the proportion of enzyme-digested fragments located in repeated sequences was as low as possible; (2) distribution of enzyme-digested fragments throughout the genome was as even as possible; (3) length of enzyme-digested fragments was in good agreement with the experimental system; (4) number of enzyme-digested fragments (SLAF tags) reached the expected number (328,885) of SLAF tags. 

### 4.5. Construction of SLAF Libraries and High-Throughput Sequencing

According to the results of the electron prediction experiment, HaeIII (New England Biolabs, Ipswich, MA, USA) was used to separately digest the eligible genomic DNA. Single-nucleotide A overhangs from these DNA fragments were polished using the Klenow fragment (New England Biolabs), and fragments were then ligated to dual-index sequencing adaptors. Adaptor-ligated fragments were then amplified by PCR, purified, pooled, and screened to construct the SLAF library [43,73]. SLAF library construction and screening were performed as described by Sun et al. (2013) [74]. From the quality-tested library, target DNA fragments of sizes from 314 to 464 bp (SLAF) were selected for paired-end sequencing on an Illumina HiSeq 2500 platform (Illumina Inc., San Diego, CA, USA) at Beijing Biomarker Technologies Corporation in Beijing, China. Additionally, we set the control genome (Oryza sativa spp. japonica; 374.30 M; http://rapdb.dna.affrc.go.jp/) to the same sequencing process, to verify whether the experimental process was reliable. The paired-end comparison efficiency of control data was 95.65%, and the enzyme digestion efficiency was 94.74%, showing that the construction of SLAF libraries was normal.

### 4.6. Identification and Distribution Analysis of Labels (SLAF Tags and SNPs)

Raw sequencing reads of SLAF were filtered for quality and trimmed to remove adaptors. The Q value was used to evaluate the single-base error rate of high-throughput sequencing. A Q value of 30 (Q30, Q < 30) represents a 0.1% error rate or 99.9% sequence accuracy. Then, the proportion of sequencing quality scores at Q_30_ in the SLAF libraries and GC content were used to evaluate the quality of sequencing. The formula for calculating the Q30 value is listed below:

(2) $$Q_{30}=-10\times \log_{10}P$$

where P is the base sequencing error rate.

Furthermore, we clustered all paired-end reads that had perfect index reads according to sequence similarity with the genome of ‘Zunla-1’. Reads with >90% identity were grouped into a single SLAF tag, and SLAF tags with a sequence that varied from sample to sample were defined as polymorphic SLAF tags [43].

Sequencing reads were aligned to the pepper reference genome (‘Zunla-1’) using BWA software; local realignments were conducted, and SNPs were detected using GATK software [75]. To ensure the accuracy of the SNPs identified using GATK, SAMtools software also was used to detect SNPs [76]. The intersection of SNPs that were detected using the two methods was designated as the final SNPs and used for further analysis. Distribution of SLAF tags and SNPs was analyzed by R (version 3.5.0, Ross Ihaka and Robert Gentleman) and SPSS software (version 22.0, International Business Machines Corporation, Armonk, New York, USA). Graphs were drawn in R (version 3.5.0, Ross Ihaka and Robert Gentleman).

### 4.7. Population Structure Analysis

Based on the filtered SNPs (integrity > 0.5; MAF > 0.05), phylogeny analysis, PCA, and population analysis were performed in turn. All of the filtered SNPs (594,429) were genotyped by MEGA6 software and used for the construction of the phylogenetic tree by the neighbor-joining algorithm [77]. PCA was performed using the smartPCA program from the EIGENSOFT package (https://github.com/DReichLab/EIG; v.6.0.1) [78]. Population structure of 287 accessions was calculated using admixture software [79]. The analysis used 594,429 SNPs of 287 accessions to infer the genetic background of an accession that belongs to a cluster under a given number of subgroups (K). The number of genetic clusters was predefined as *K* = 1–15 for all accessions to explore the population structure. The relatedness coefficient was counted using SPAGeDi (version 1.4C) software [80]. Linkage disequilibrium between SNPs was estimated with R^2^ using HAPLOVIEW software (https://www.broadinstitute.org/ftp/pub/mpg/haploview/hapinstall.exe) [81].

### 4.8. Genome-Wide Association Analyses of 36 Agronomic Traits

Total filtered SNPs (integrity > 0.5; MAF > 0.05) detected from 287 accessions were used for GWAS. GWAS for all traits (based on GLM, MlM, CMLM, FaST-LMM, and EMMAX models) was conducted using TASSEL (http://www.maizegenetics.net/tassel), FaST-LMM (https://www.microsoft.com/en-us/download/confirmation.aspx?id=52588), and EMMAX (http://csg.sph.umich.edu//kang/emmax/download/index.html) software with default settings [82,83,84]. Details of those model formulae are as follows:

The general linear model formula was:

(3) y=Xα+Qβ+e

The mixed linear model formula was:

(4) y=Xα+Qβ+Kμ+e

where y is the phenotype (for qualitative traits, phenotypic data were the same for 2017, 2018 and 2019; for quantitative traits, it was the mean of nine replications over three years, as shown in Table S2), X is the genotype, Q is the Q matrix, and K is the relative relatedness matrix. Xα and Qβ are fixed effects, and Kμ and e are random effects. The Q value, which represents population structure, was calculated by the admixture software [79]. The K value, which represents the relative relatedness between accessions, was calculated by the SPAGeDi software (version 1.4C) [80].

The *p* value was calculated for each SNP, and *p* < 1.707 × 10^−8^ (*p* = 0.01/*n*; *n* = total markers used, which is roughly a Bonferroni correction, corresponding to −log10 (*p*) = 8, blue line) and *p* < 1.707 × 10^−7^ (*p* = 0.1/*n*; *n* = total markers used, which is roughly a Bonferroni correction, corresponding to −log10 (*p*) = 7, red line) was defined as the suggestive (highly significant) threshold and genome-wide control (significant) threshold, respectively. The genes within 100 kb up- or down-stream of these significant SNPs were found by Jbrowser in SGN (Sol Genomics Network https://solgenomics.net/) and reported as potential candidate genes [46,47]. The Manhattan and QQ plots of GWAS were drawn using the R package “qqman.”. The heatmaps of the SNPs and genes number in pairwise traits and the physical map of significant SNPs (or polymerization regions) associated with 26 traits were drawn by R software (version 3.5.0, Ross Ihaka and Robert Gentleman).

### 4.9. Molecular Mapping of the Nuclear Fertility-Restoring Gene (Rf) for Cytoplasmic Male Sterility

Two F_2_ populations were constructed separately in spring 2016 (334 individuals) and spring 2017 (5026 individuals) for fine mapping, with parents (17C641 and 17C643) selected from the GWAS population. In these two populations, the 2016 population was used for initial mapping, and the 2017 population was subsequently used for fine mapping. All individuals were grown inside greenhouses with an inter-individual distance of 30 cm, and unified field management was used. Methodological details are provided in Note S1.

A genotypic assay was performed using SSR markers (Table S20). The SSR markers that cover the entire pepper genome were designed according to the published pepper genome sequence. The SSR loci that covered the entire genome were developed using a whole *C. annuum* ‘Zunla-1’ genome scan of double-base repeats, and SSR loci that were repeated six times or more developed using misa.pl.300bp software were extended using Python scripts on each side of the SSR loci. The primers were designed using Primer 3 (Premier Biosoft International, Palo Alto, CA, USA). The PCR solution was 10 μL in volume and contained 1.0 μL DNA template, 0.5 μL of both forward and reverse primers, 5.0 μL Taq polymerase mix (Beijing ComWin Biotech Co.,Ltd., Beijing, China), and 3 μL ddH_2_O. The PCR solution was 10 μL and contained 1.0 μL DNA template, 0.5 μL of both forward and reverse primers, 5.0 μL Taq polymerase mix, and 3 μL ddH_2_O. The PCR protocol was as follows: initial denaturation at 94 °C for 5 min; 35 cycles of denaturation at 94 °C for 30 s, annealing at 53 °C for 45 s, and extension at 72 °C for 30 s, before a final extension at 72 °C for 5 min. PCR products were stored at 4 °C and analyzed using 7% polyacrylamide gel electrophoresis. Joinmap 4.0 software (Beijing Lucidus Bioinformation Technology Co., Ltd., Beijing, China) was used to analyze the linkage relationship between the markers and the *Rf* gene. RT-PCR, real-time PCR, and sequencing of PCR products were performed as described by Wang et al. (2019) [85].

## 5. Conclusions

Based on the SLAF-seq, the GWAS of 36 agronomic traits in 287 pepper accessions was performed in this study. A total of 1,824,874 SLAF tags, 1,025,395 polymorphism SLAF tags and 9,557,790 SNPs, were obtained. Those tags/labels were normally distributed in the population consisted of 287 peppers. Meanwhile, those tags/labels were uniformed distributed on the 12 pepper chromosomes, indicating those SLAF tags and SNP developed can be used for GWAS. In order to obtain a better result, Fast-LMM, EMMAX and GLM were selected as the optimal statistical models for certain traits. In our study, a total of 1487 SNP, 2126 candidate genes and 109 correlation peaks were significantly (*p* < 1.707 × 10^−8^) correlated with the 26 agronomic traits in pepper by GWAS. Thirty-seven SNP polymerization regions were obtained from the correlation analysis. The two traits with higher correlation index may locate in a same SNP polymerization region. These results is useful for multi-trait pyramid breeding of pepper. In addition, based on the gene annotation and expression patterns, *Capana06g002967* and *Capana06g002969* were identified as the candidates for the *Rf* gene, which provided a reference for the further study of CMS in *Capsicum*.

## Figures and Tables

**Table 1:** Details of peak regions associated with different traits identified via a genome wide association study (GWAS) in pepper.

Plant Organ	Trait	Model	Chromosome	Number of SNPs (*p* < 1.707 × 10^−8^) in Peak Region	Trait-Associated Peak Region		
					Start	End	Size (Mbp)
Stem	Pt	FaST-LMM	Chr01	9	278425275	280417460	1.992185
			Chr04	2	23932585	23972683	0.040098
			Chr05	5	17744799	19221034	1.476235
			Chr06	10	5675663	5759294	0.083631
			Chr09	10	12510495	19373800	6.863305
			Chr09	14	118192571	119757960	1.565389
			Chr09	10	171029237	173134444	2.105207
			Chr11	3	37287718	40113909	2.826191
			Chr11	17	107259335	107501665	0.24233
	Bh	EMMAX	Chr01	59	275555035	277575138	2.020103
			Chr02	6	83643192	85422044	1.778852
			Chr02	21	104238462	104524382	0.28592
			Chr03	13	253213778	254639900	1.426122
			Chr06	14	13971060	18672334	4.701274
			Chr07	9	5373860	7707856	2.333996
			Chr07	35	184612786	184825374	0.212588
			Chr09	10	191839433	200002779	8.163346
	Msc	FaST-LMM	Chr06	2	20231877	20231907	0.00003
			Chr08	6	114388151	114431559	0.043408
			Chr09	2	214330476	214330504	0.000028
	Msp	EMMAX	Chr11	28	27131483	28706358	1.574875
Leaf	Ls	GLM	-	-	-	-	-
	Lc	GLM	-	-	-	-	-
	Lm	GLM	Chr01	8	158420891	158729605	0.308714
			Chr04	7	58610574	59222198	0.611624
			Chr06	7	212371837	212424874	0.053037
			Chr08	7	131427437	134733324	3.305887
			Chr12	4	12354265	19260025	6.90576
	Lp	EMMAX	Chr10	9	162170912	162221229	0.050317
			Chr10	4	197927729	200867123	2.939394
			Chr12	3	22681366	22692847	0.011481
	Lsc	GLM	-	-	-	-	-
Flower	Cc	EMMAX	Chr02	4	68060203	68060282	0.000079
			Chr04	6	189062369	192962061	3.899692
			Chr06	8	41630661	41857768	0.227107
			Chr07	2	214559213	214585881	0.026668
			Chr07	6	220688885	220941545	0.25266
			Chr08	8	135096972	135952487	0.855515
			Chr09	5	201471658	202362543	0.890885
			Chr11	5	28177723	28191389	0.013666
			Chr11	6	214289893	216304968	2.015075
	Sc	GLM	Chr01	3	211183454	211183884	0.00043
			Chr10	24	155035157	155097491	0.062334
			Chr11	114	27555236	29305711	1.750475
	Ac	FaST-LMM	Chr01	17	246812579	249477491	2.664912
			Chr07	2	130948617	130973590	0.024973
	Ffn	FaST-LMM	-	-	-	-	-
	Nfpa	EMMAX	Chr01	3	220458255	220462790	0.004535
			Chr01	7	280054497	280417460	0.362963
			Chr04	3	57766474	58558409	0.791935
			Chr05	2	206654389	206654579	0.00019
			Chr06	10	13971060	19735646	5.764586
			Chr06	39	219098248	219470017	0.371769
			Chr07	3	185249295	185271463	0.022168
			Chr09	22	10463866	12820022	2.356156
			Chr09	12	118192571	119641888	1.449317
			Chr10	7	29037048	33183732	4.146684
			Chr11	5	209423789	210867712	1.443923
			Chr12	20	192018036	193789860	1.771824
	Fpgs	EMMAX	Chr12	23	32930746	38073141	5.142395
	Ms	FaST-LMM	Chr06	101	214443037	215536517	1.09348
Fruit	Aif	EMMAX	Chr08	4	130745390	131501395	0.756005
			Chr09	6	104816188	104816287	0.000099
			Chr09	5	167226310	167226390	0.00008
			Chr10	86	148782468	155625398	6.84293
	Fsf	EMMAX	-	-	-	-	-
	Fg	FaST-LMM	Chr07	8	6280167	6685034	0.404867
			Chr09	3	39263	1201526	1.162263
			Chr12	8	180112718	186338088	6.22537
	Fsc	EMMAX	-	-	-	-	-
	Fss	FaST-LMM	Chr01	3	202642462	203121398	0.478936
			Chr04	2	15454448	15454730	0.000282
			Chr09	4	94667918	94693832	0.025914
			Chr09	8	126292337	127360615	1.068278
			Chr11	9	30762829	49631336	18.868507
	Fas	FaST-LMM	-	-	-	-	-
	Ab	FaST-LMM	-	-	-	-	-
	Mfc	EMMAX	Chr06	17	3758563	10060744	6.302181
	St	FaST-LMM	Chr04	2	3047655	3280037	0.232382
			Chr05	2	54372525	54372671	0.000146
			Chr06	3	40301536	40301894	0.000358
			Chr10	3	201908626	203356884	1.448258
			Chr12	3	6307012	12354265	6.047253
			Chr12	45	35222952	35714999	0.492047
	Fpl	FaST-LMM	-	-	-	-	-
	Pw	FaST-LMM	Chr08	4	133185108	133433188	0.24808
			Chr12		203552366	206591342	3.038976
	Pl	FaST-LMM	-	-	-	-	-
	Psi	FaST-LMM	-	-	-	-	-
	Fw	FaST-LMM	Chr01	4	103860245	105083614	1.223369
			Chr08	22	133045048	133584887	0.539839
			Chr12	9	201671262	206591342	4.92008
	Fl	FaST-LMM	-	-	-	-	-
	Fsi	EMMAX	-	-	-	-	-
	Wpf	FaST-LMM	Chr01	7	103860245	105083614	1.223369
			Chr05	2	168537584	168540175	0.002591
			Chr08	4	133396929	133433188	0.036259
			Chr12	14	200798023	211814051	11.016028
	Dmc	FaST-LMM	Chr02	7	69956806	79414868	9.458062
			Chr11	5	85204434	85204791	0.000357
	Tf	FaST-LMM	Chr01	3	103860245	105083388	1.223143
			Chr08	3	126042291	133396929	7.354638
			Chr11	5	128979925	128979983	0.000058
			Chr12	8	200798023	207472045	6.674022
	Nol	FaST-LMM	-	-	-	-	-

Note: Pt, plant type; Bh, branching habit; Msc, main stem color; Msp, main stem pubescence; Ls, leaf shape; Lc, leaf color; Lm, leaf margin; Lp, leaf pubescence; Lsc, leaf surface characteristics; Cc, corolla color; Sc, style color; Ac, anther color; Ffn, first flower node; Nfpa, number of flowers per axil; Fpgs, flower pedicel growing state; Ms, Male-sterility; Aif, anthocyanin on immature fruit; Fsf, fruit surface furrow; Fg, fruit glossy; Fsc, fruit surface characteristics; Fss, fruit shoulder shape; Fas, fruit apex shape; Ab, appendage at blossom-end; Mfc, mature fruit color; St, spicy type; Fpl, fruit pedicel length; Pw, placenta width; Pl, placenta length; Psi, placenta size index; Fw, Fruit width; Fl, fruit length; Fsi, fruit shape index; Wpf, weight per fruit; Dmc, dry matter content; Tf, thickness of flesh; Nol, Number of locules (full text identical). Bold font represents the 13 quantitative traits classified by data type. ‘-’, which indicated that there were no significant peaks.

## Supplementary Materials

Supplementary Materials can be found at https://www.mdpi.com/1422-0067/20/22/5675/s1.
