# Supplementary material for: Genome-Wide Correlation of 36 Agronomic Traits in the 287 Pepper (Capsicum) Accessions Obtained from the SLAF-seq-Based GWAS
Source: Int J Mol Sci. 2019 Nov 13;20(22):5675. doi: 10.3390/ijms20225675 (PMC6888518; doi:10.3390/ijms20225675)
Supplement: Supplementary file 1 [file ijms-20-05675-s001.zip › ijms-611185-proofreading-si/supplementary material/Supplementary_Material0.pdf]

International Journal of Molecular Sciences
MDPI

Supplementary Material

1 Supplementary Note, Figures, Gif, and Tables

1.1 Supplementary Note

Supplementary Note 1: Survey of 36 agronomic traits in 287 pepper accessions

A total of 36 agronomic traits were investigated in our study, which related to the characteristics of four organs, including stem (4), leaf (5), flower (7) and fruit (20). Three to six plants of each accession were selected to survey the 36 agronomic traits. The surveying method for each trait was according to *Descriptors and Data Standard for Capsicum (Capsicum annum L., Capsicum frutescens L., Cpasicum chinense, Capsucum baccatum, Capsicum pubescens*) (Li et al., 2006) with some slight modifications. Here, details of the surveying methods for 36 agronomic traits were listed below:

The four stem-related traits

Plant type: The measurement was conducted when the flowers on the third branch opened.The angle between branch and the main stem branch was measured with a protractor. Based on the branch angle, the plant type could be divided into three classes: spreading (＞90°), semi-erect (45°~90°) and erect (＜45°). (**Supplementary Figure 1a**).

Branching habit: The measurement was conducted when the flowers on the first branch opened.The growth situation and the emerging of the axillary buds were recorded. Based on the axillary buds, the branching habit was classified into two types: determinate type (shoot apical meristem (SAM) transferred into floral meristem (FM) which leadsself-pruning, **supplementary Figure 1b.**) and indeterminate type (after SAMtransferred into FM, an axillary SAM emerged atthe below leaf axil,**supplementary Figure 1c**).

Main stem color: This trait was investigated using the RHS colorimetric card at the time when the flowers on the third branch opened **(Supplementary Figure 1d).**

Main stem pubescence: The investigation was conducted when the flowers on the third branch opened. The density of the pubescence was classified into four types: none, sparse, intermediate and dense **(Supplementary Figure 1e).**

The five Leaf-related traits

Leaf-lelated traits including leaf shape, leaf color, leaf margin, leaf pubescence and leaf surface characteristic were investigated in the leaves on the second branch when the flowers on the third branch opened

Leaf shape, leaf margin and leaf surface characteristics: The adaxial surface of the leaf was scanned using a scanner (BENQ 5560) and analyzed using ImageJ software. Leaf shape, leaf margin and leaf surface characteristics were classified according to **supplementary figure 2a,c,e.** Particularly, for leaf surface characteristics, the shrinkage degree of adaxial side of leaf was investigated.

Leaf color: Color of the adaxial side of the leaf was measured using a RHS colorimetric card **(supplementary figure 2b).**

Leaf pubescence: This trait was investigated on the adaxial side of the leaf. Classification of this trait was based on the density of pubescence **(Supplementary Figure 2d)**.

The seven flower-related traits

Corolla color, style color, anther color and first flower node were investigated when the first flower opened. For each plant, three to six flowers were investigated and for each accession, three to six plants were investigated.

Corolla color, style color and anther color: Those traits were measured under a normal and consistent illumination condition using a RHS colorimetric card **(Supplementary Figure 3a,b).**For anther color, anthers were collected from the flowers and the measurements were conducted on a while background.

First flower node: This trait was defined as the true leaf number on the stem below the first flower.

Besides the traits mentioned above, flower pedicel growing state, male-sterility and number of flowers per axil were also investigated.

Flower pedicel growing state: This type of trait was investigated when flowers on the second branch opened. The flower pedicel growing stage was defined as the angle between pedicel and stem which was measured using a protractor.

Based on the angle, the flower pedicel growing state can be classified into three types: pendant (＞105°), horizontal (75°~105°) and erect (＜75°). **(Supplementary Figure 3 c,d,e)**

Male-sterility (*Rf*):

For GWAS populations, Whether an accession harboring the *Rf* gene or not was determined previously by crossing each accession with the CMS line.

For F_2 populations, The male fertility of each plant was investigated at anthesis stage. A male fertile flower must meet the following conditions: flowers are full of stamens; the anthers are filled with pollen. The fertility was further verified based on fruit set rate as well as the fruit characteristics. Particularly, the fruits derived from the self-pollinated male sterile flowers are small and parthenocarpic.

Number of flowers per axil: Three to six plants were randomly selected from each accession and the number of flowers of each axil at the second branch was visually investigated (**supplementary Figure 3f-j).**

The 20 fruit-related traits

Anthocyanin on immature fruit: Immature fruit color was visually inspected **(Supplementary Figure 4a)**

Fruit surface furrow and fruit surface characteristics: The depth of furrow and the shrinkage of the fruit were visually inspected. Classification standards for those two traits were established based on the investigation of all the accessions.

Fruit glossy, fruit shoulder shape, fruit apex shape and appendage at blossom-end: The fruit glossy, fruit shoulder shape, fruit apex shape and appendage at blossom-end were visually inspected. Fruit shoulder shape, apex shape and appendage at blossom-end were classified according to **supplementary figure 4b,c,e,** respectively.

Fruit pedicel length, fruit size and placenta size (width, length and size index): Fruits were cut longitudinally and scanned with a scanner (BENQ 5560). Fruit pedicel length, fruit size and placenta size were measured using ImageJ software**(Supplementary Figure 4f).** Fruit size index= fruit length / fruit width; placenta size index= (placenta width×placenta length) / (fruit width×fruit length))

Thickness of flesh and number of locules: Fruits were cut longitudinally and scanned with a scanner (BENQ 5560). Thickness of flesh and number of locules were measured using ImageJ software.

Spicy type: This trait was measured by tasting.

Dry matter content and weight per fruit: Three to six fruits on the second branch were collected from each accession. Dry matter content=(weight before drying-weight after drying)/weight before drying; fresh weight per fruit= total fresh weight before drying/number of fruits.

Mature fruit color: This trait was investigated using a RHS colorimetric card when the flower on the second branch opened **(Supplementary Figure 4d).**

1.2 Supplementary Figures

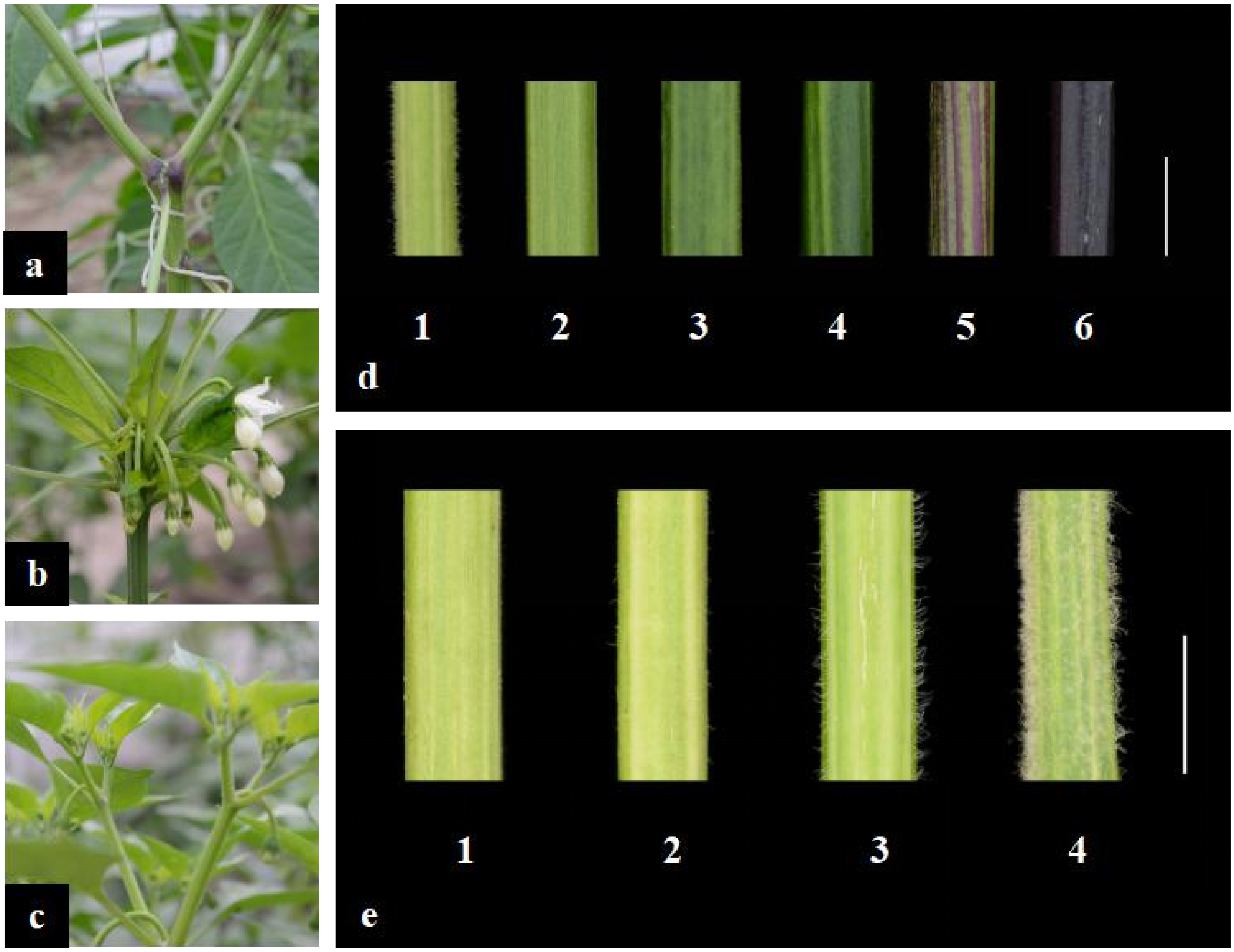

Supplementary Figure 1: Phenotypic types of stem-related agronomic traits in 287 pepper accessions.a, angle of first branch of main stem of pepper; b, terminal bud morphology of determinate branching; c, terminal bud morphology of indeterminate branching types; d, Color of main stem of pepper: 1, yelloish green; 2, light green; 3, green; 4, dark green; 5, green with purple stripe; 6, purple; e, main stem pubescence:1, none; 2, sparse; 3, intermediate; 4, dense. The white bar represents 1 cm.

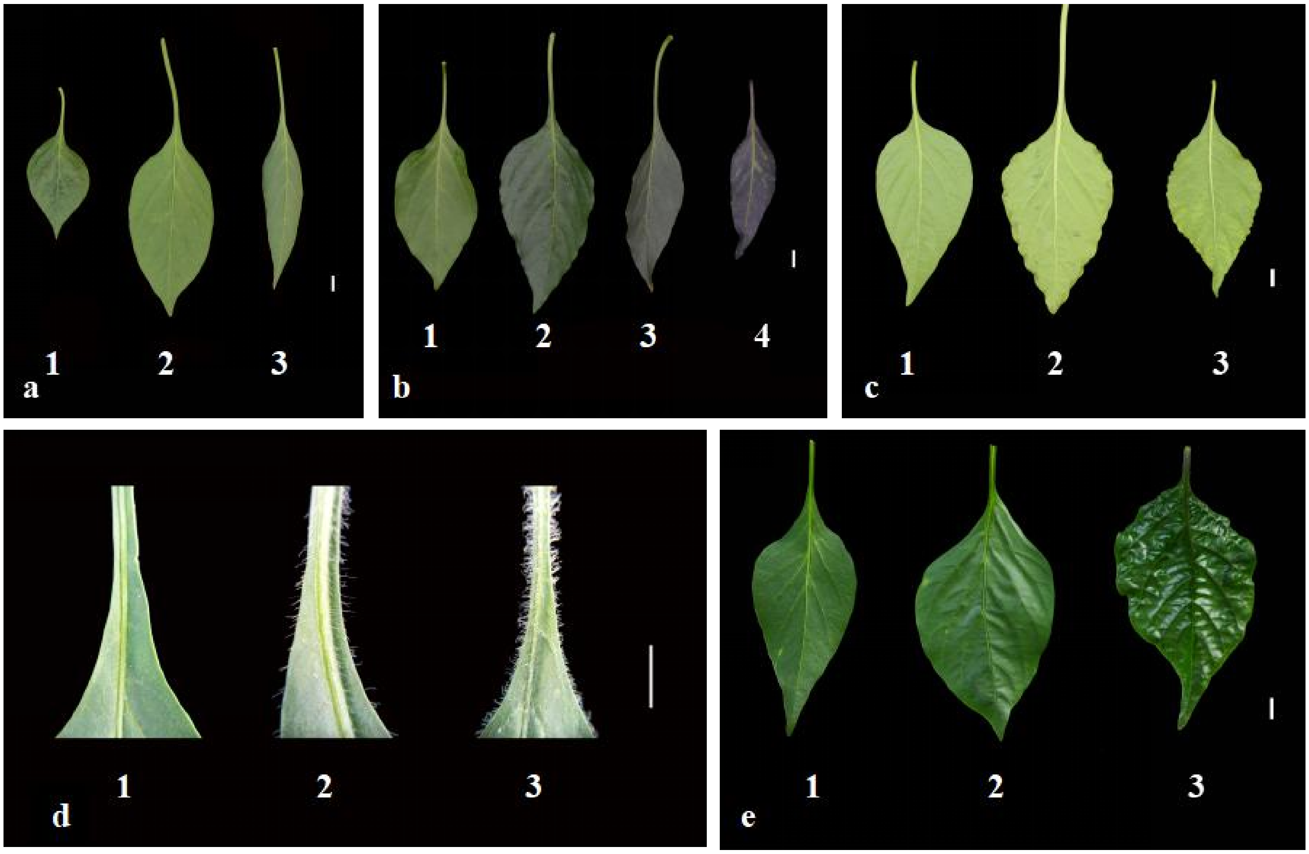

Supplementary Figure 2: Phenotypic types of leaf-related agronomic traits in 287 pepper accessions.a, leaf shape: 1, obvate; 2, long obvate; 3, lanceolate; b, leaf color: 1,light green; 2, green; 3, dark green; 4, green with purple; c, leaf magin: 1, entire; 2, undulate; 3, serrate; d, leaf pubescence: 1, none; 2, intermediate; 3, dense; e, leaf surface characteristics: 1, smooth 2, slightly wrinkling; 3, wrinkling. The white bar represents 1 cm.

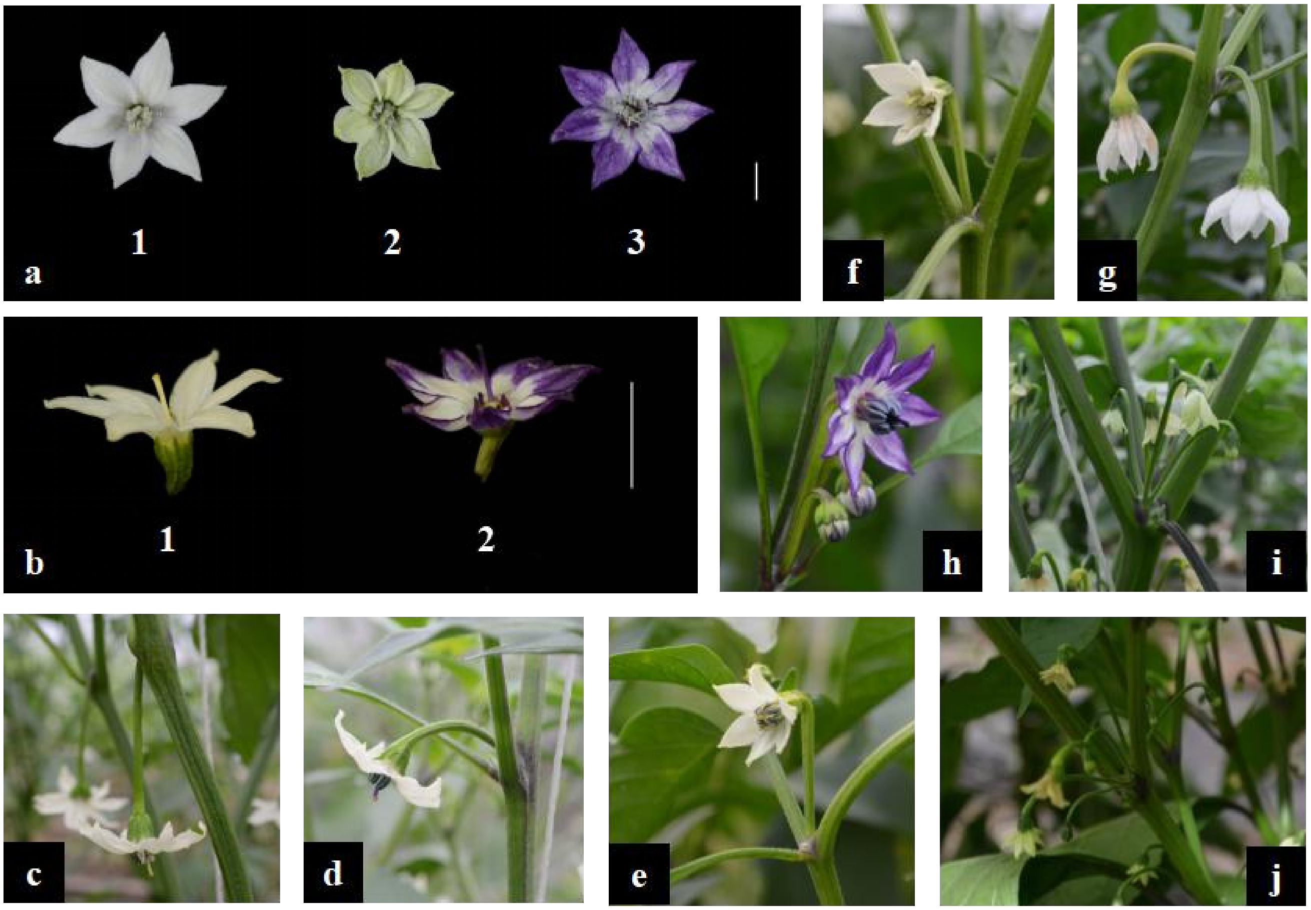

Supplementary Figure 3: Phenotypic types of flower-related agronomic traits in 287 pepper accessions.a, corolla color: 1, white; 2, light green; 3, purple; b, style color: 1, white; 2, purple; c, pendant flower pedicel; d, horizontal flower pedicel; e, erect flower pedicel; f, g, h, i and j: number of flowers per axil. The white bar represents 1 cm.

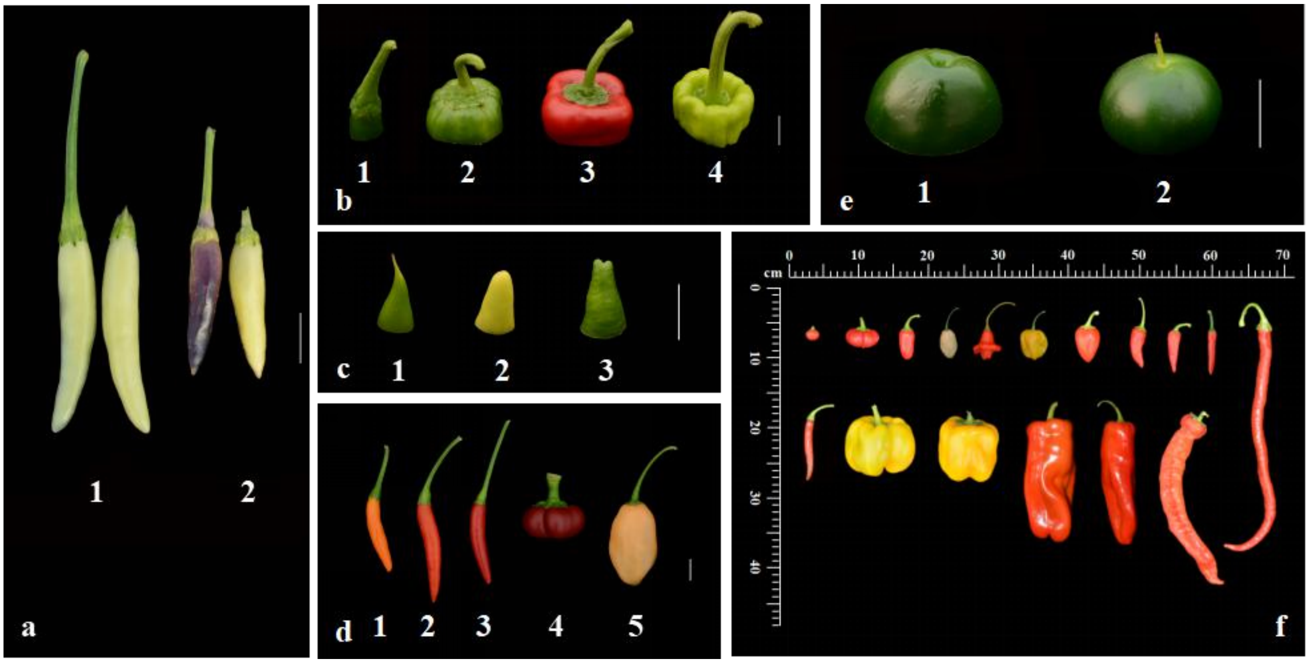

Supplementary Figure 4: Phenotypic types of fruit-related agronomic traits in 287 pepper accessions.a, anthocyanin on immature fruit: 1, none; 2, present; b, fruit shoulder shape: 1, none; 2, nearly flat; 3, sunken; 4, deep sunken. c, fruit apex shape: 1, pointed; 2, blunt; 3, sunken; d, mature fruit color: 1, orange; 2, tangerine; 3, bright red; 4, dark red; 5, chocolate yellow; e, appendage at blossom-end: 1, none; 2, present; f, various size pepper fruits; The white bar represents 1 cm.

a

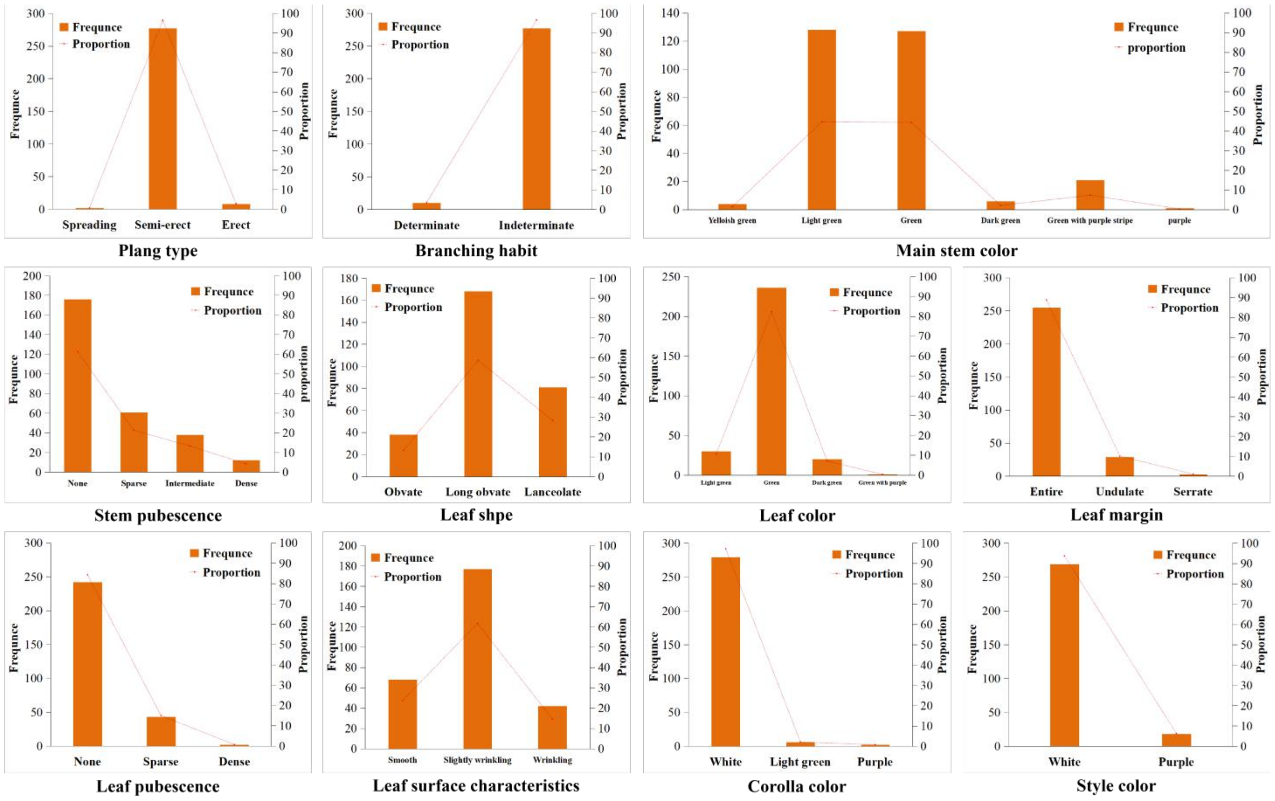

Supplementary Figure 5: Distribution of 23 qualitative agronomic traits in 287 pepper accessions (a).

b

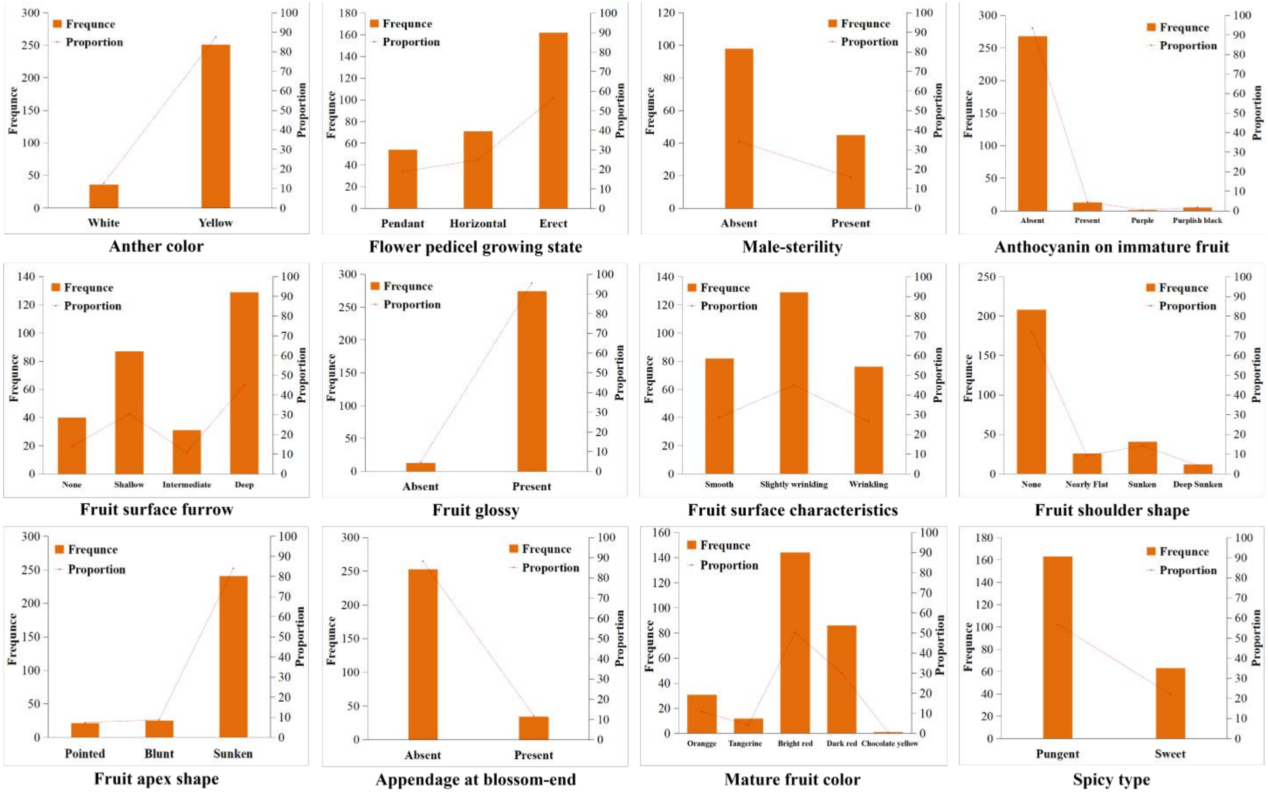

Supplementary Figure 5: Distribution of 23 qualitative agronomic traits in 287 pepper accessions (b).

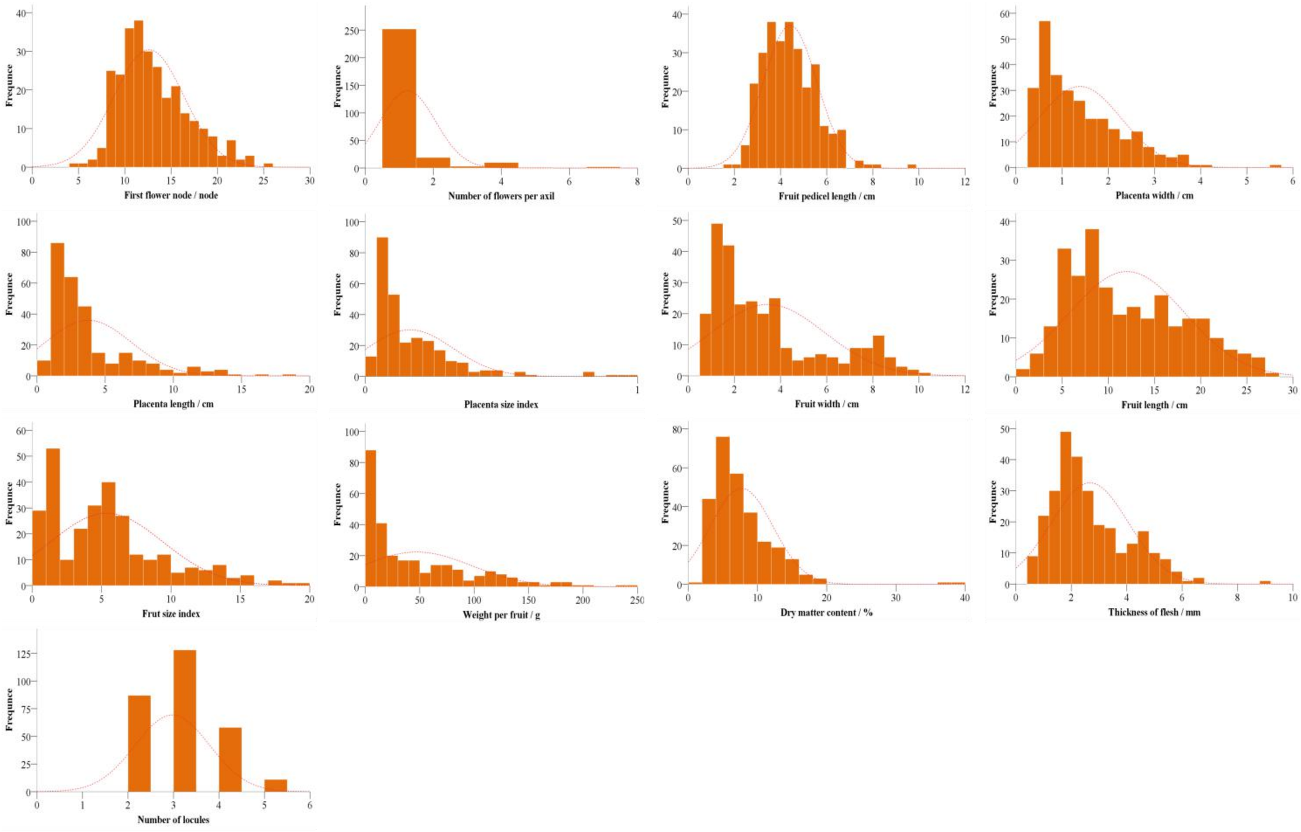

Supplementary Figure 6: Distribution of 13 quantitative agronomic traits in 287 pepper accessions.

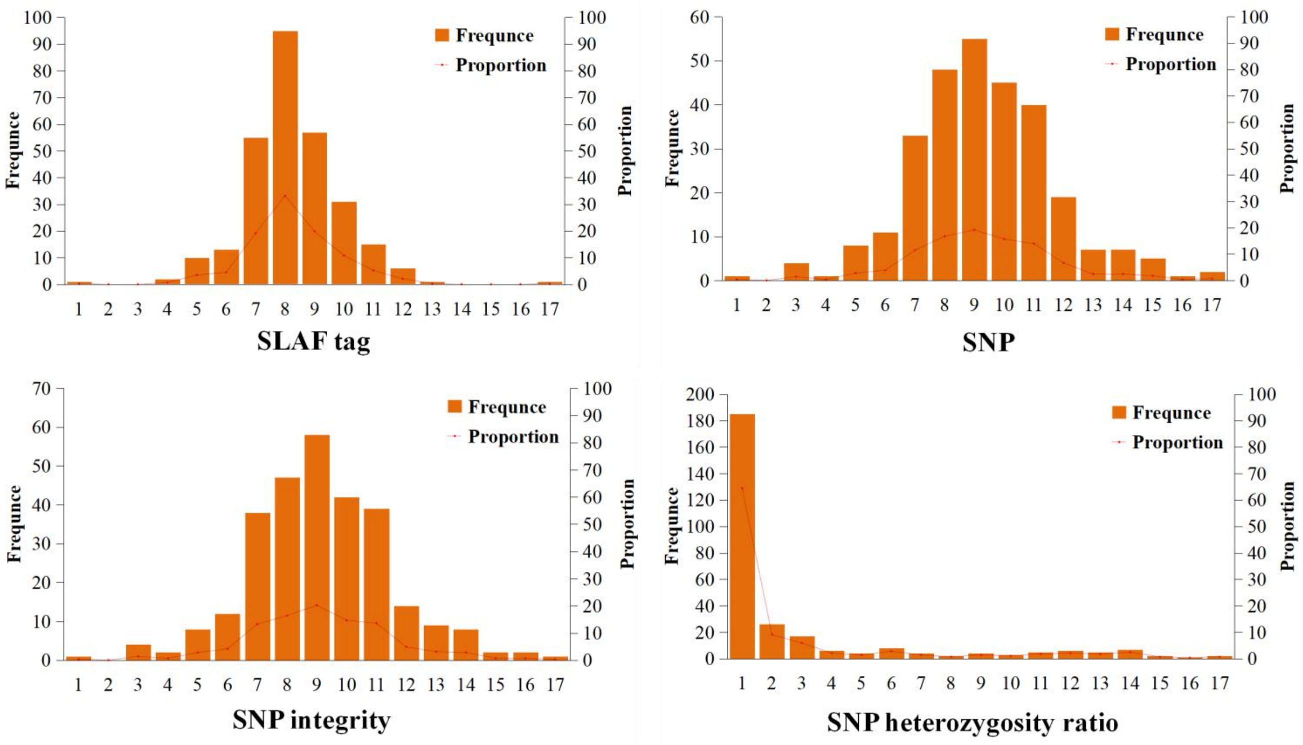

Supplementary Figure 7: Distribution of SLAF tags, SNPs, SNPs integrity and SNPs heterozygosity in 287 pepper accessions.

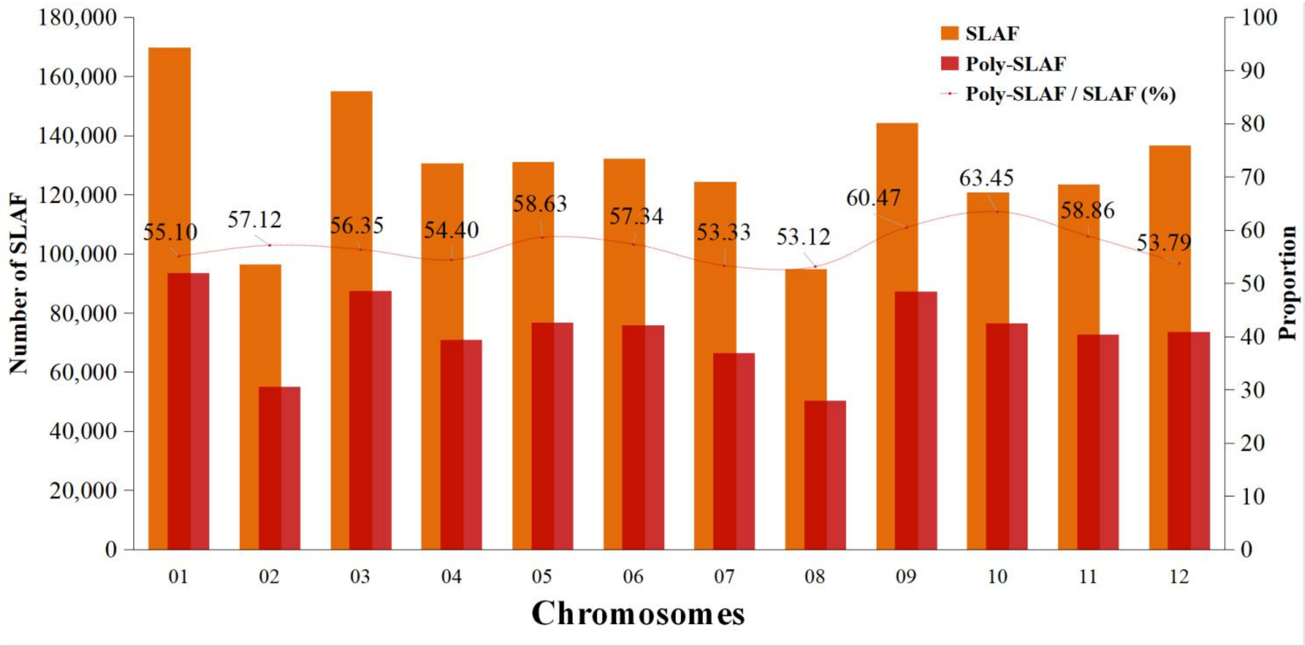

Supplementary Figure 8: Distribution of SLAF and ploy-SLAF on chromosomes.

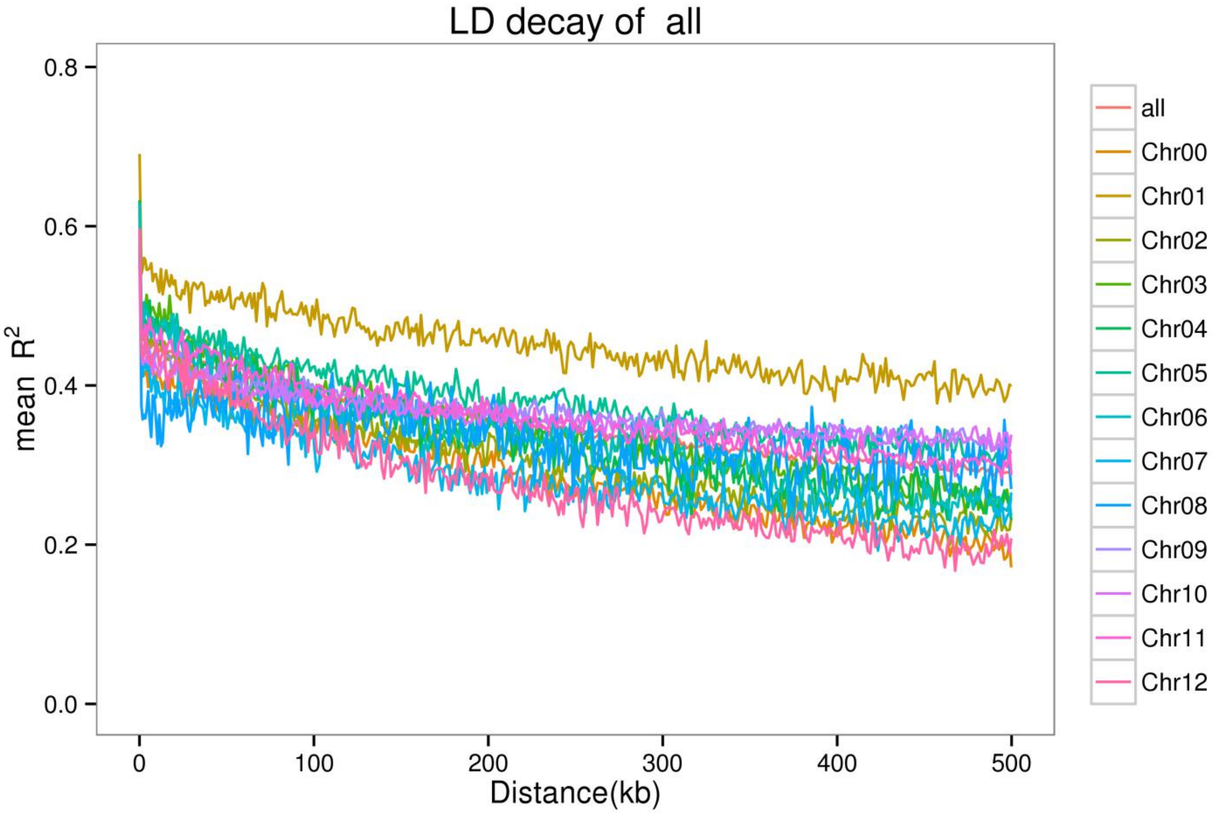

Supplementary Figure 9: Linkage disequilibrium decay determined according to squared correlations of allele frequencies (R^2).

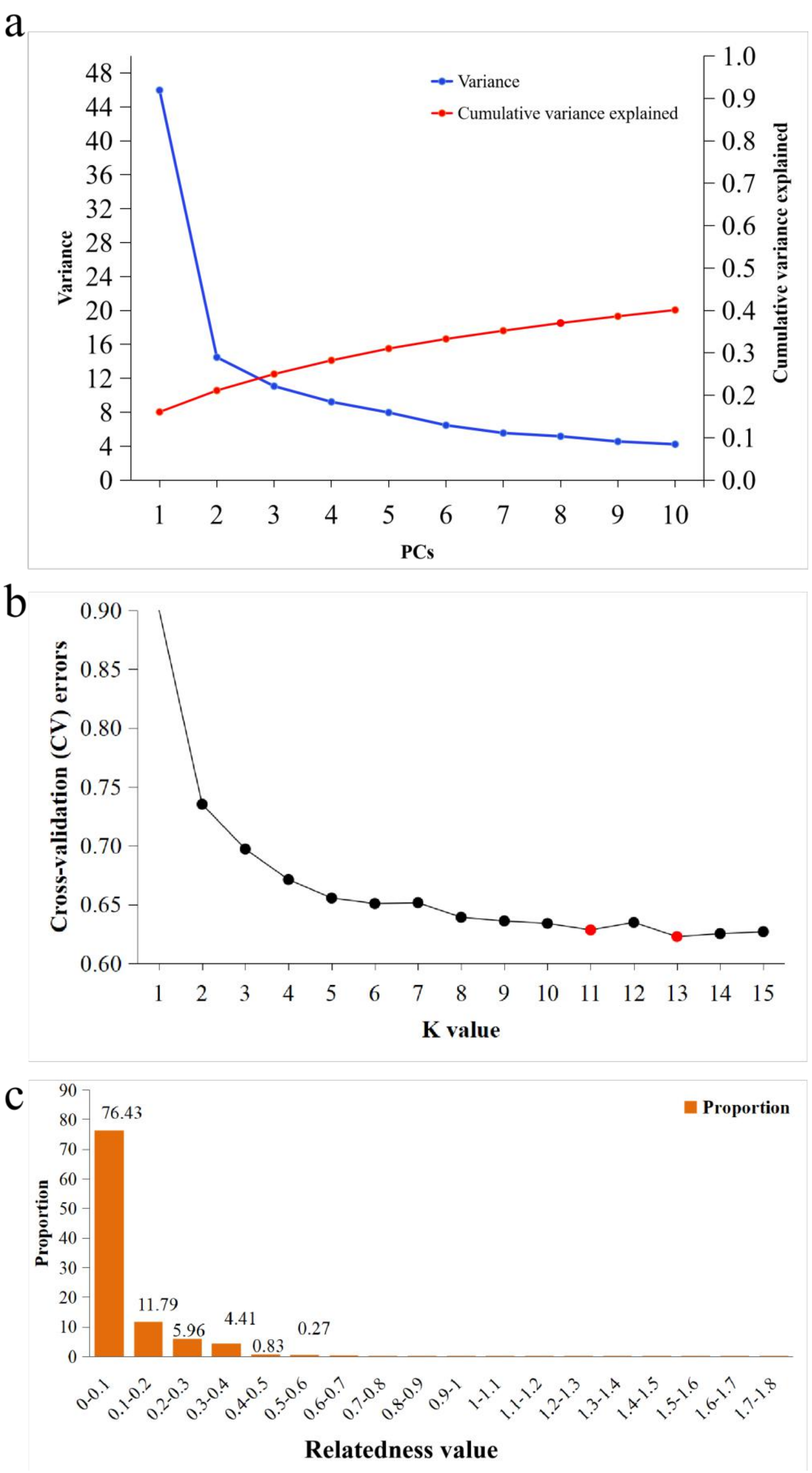

Supplementary Figure 10: Population structure analysis for 287 pepper accessions.a. Principal component analysis of 287 pepper accessions. b. K values plotted from 1 to 13. c. Histogram frequency distribution of pairwise relative relatedness coefficients.

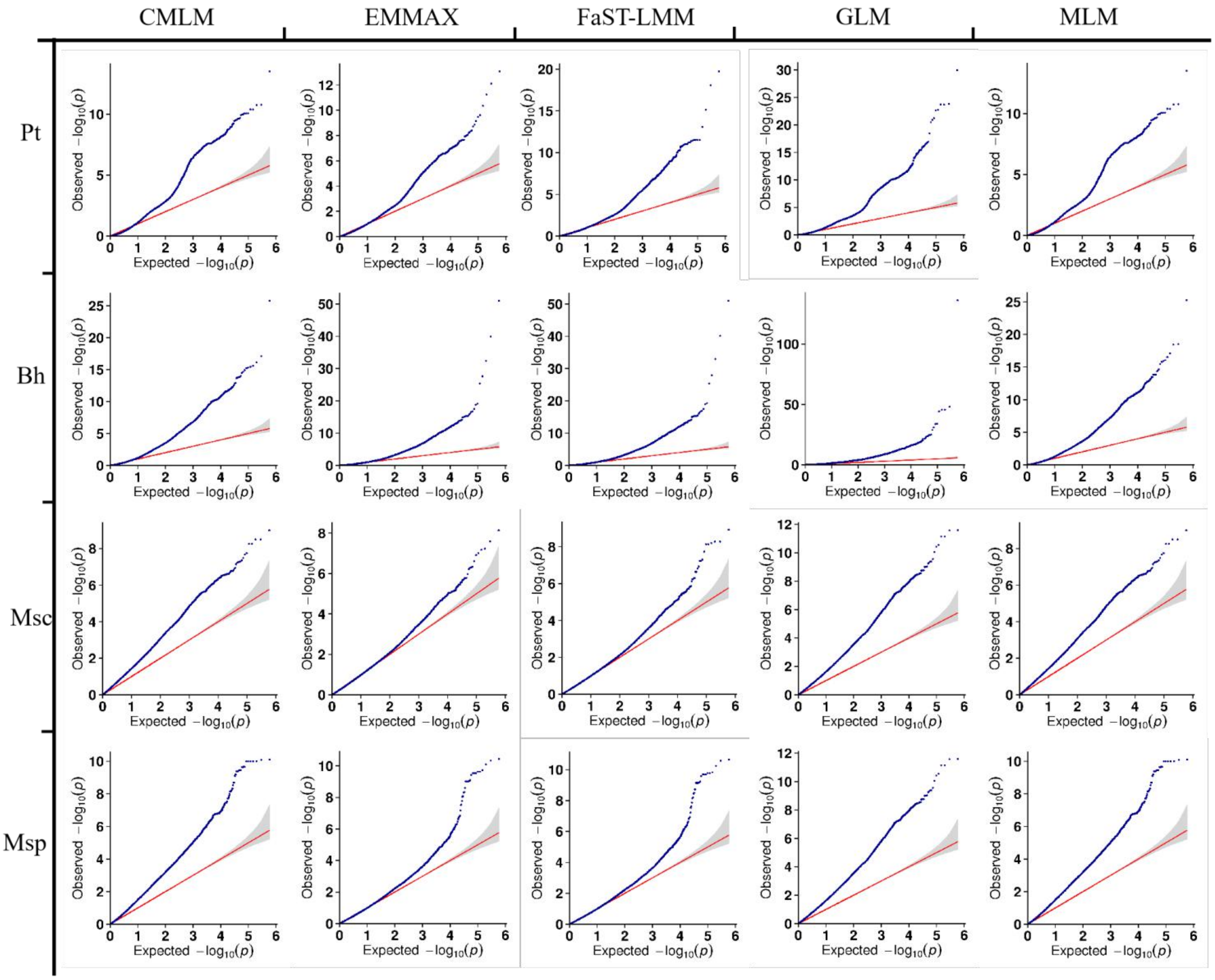

Supplementary Figure 11:Q-Q plots of stem-related agronomic traits.Pt, plant type; Bh, branching habit; Msc, main stem color; Msp, main stem pubescence.

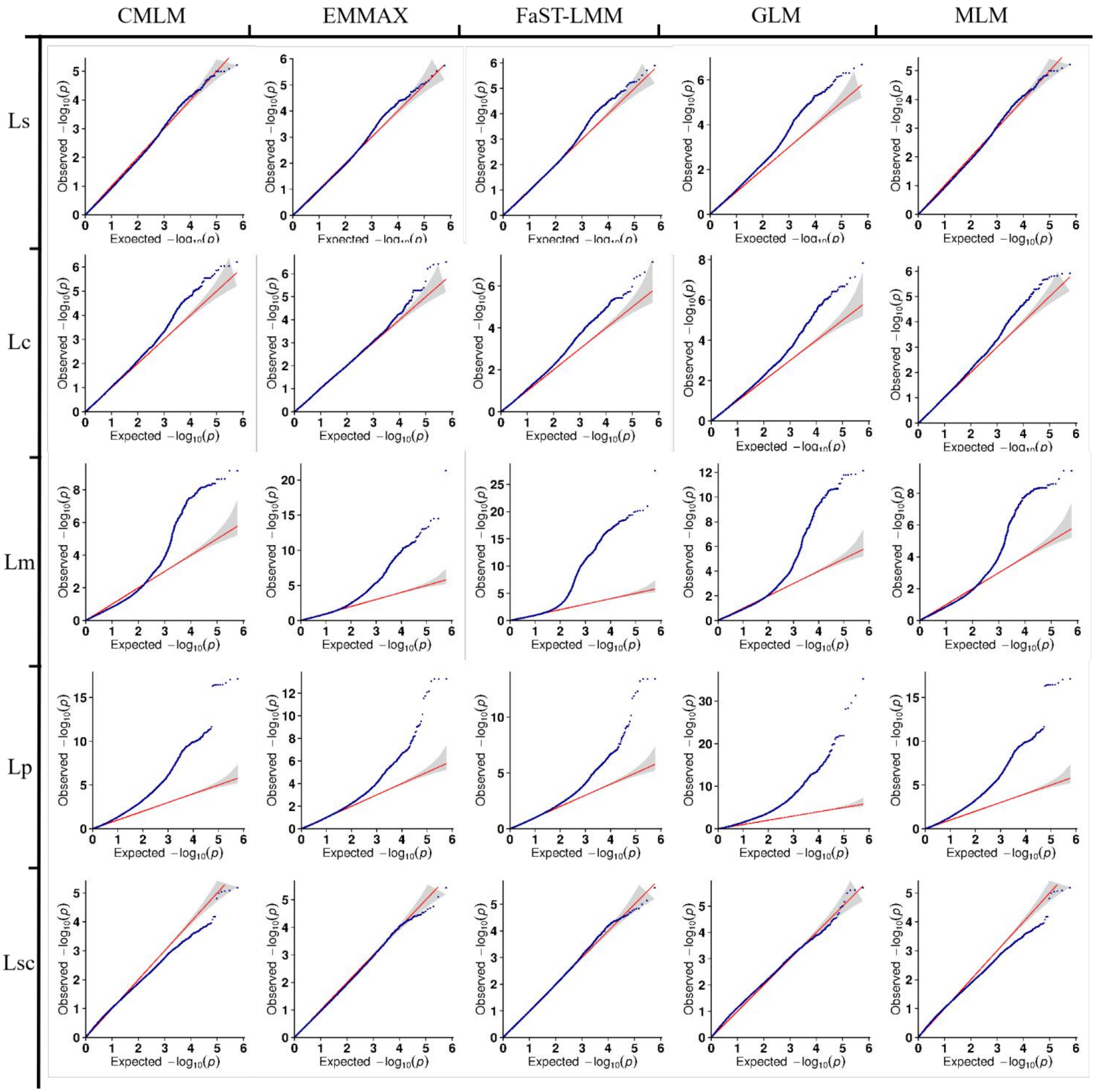

Supplementary Figure 12: Q-Q plots of leaf-related agronomic traits. Ls, leaf shape; Lc, leaf color; Lm, leaf margin; Lp, leaf pubescence; Lsc, leaf surface characteristics.

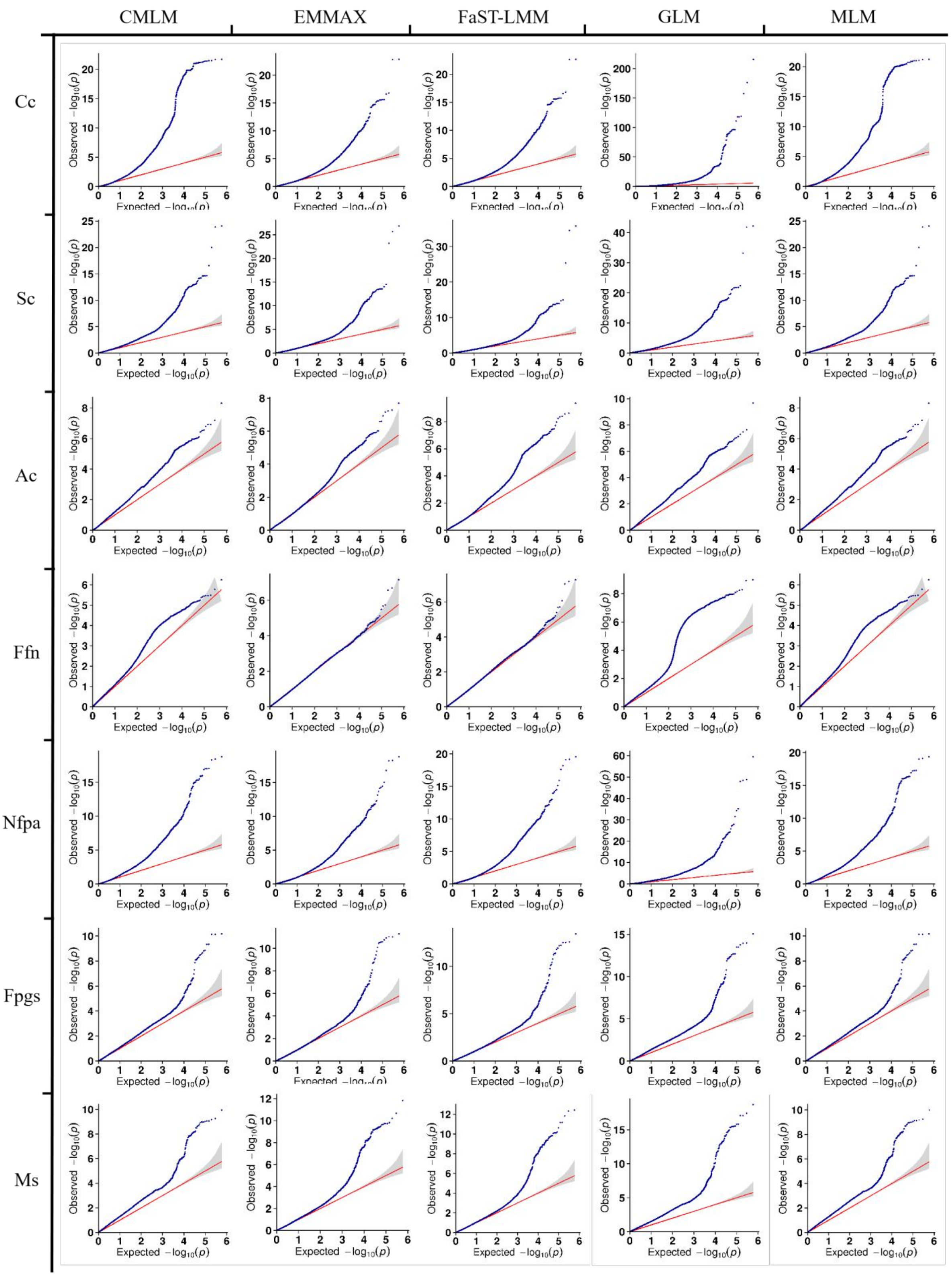

Supplementary Figure 13: Q-Q plots of flower-related agronomic traits. Cc, corolla color; Sc, style color; Ac, anther color; Ffn, first flower node; Nfpa, number of flowers per axil; Fpgs, flower pedicel growing state; Ms, Male-sterility.

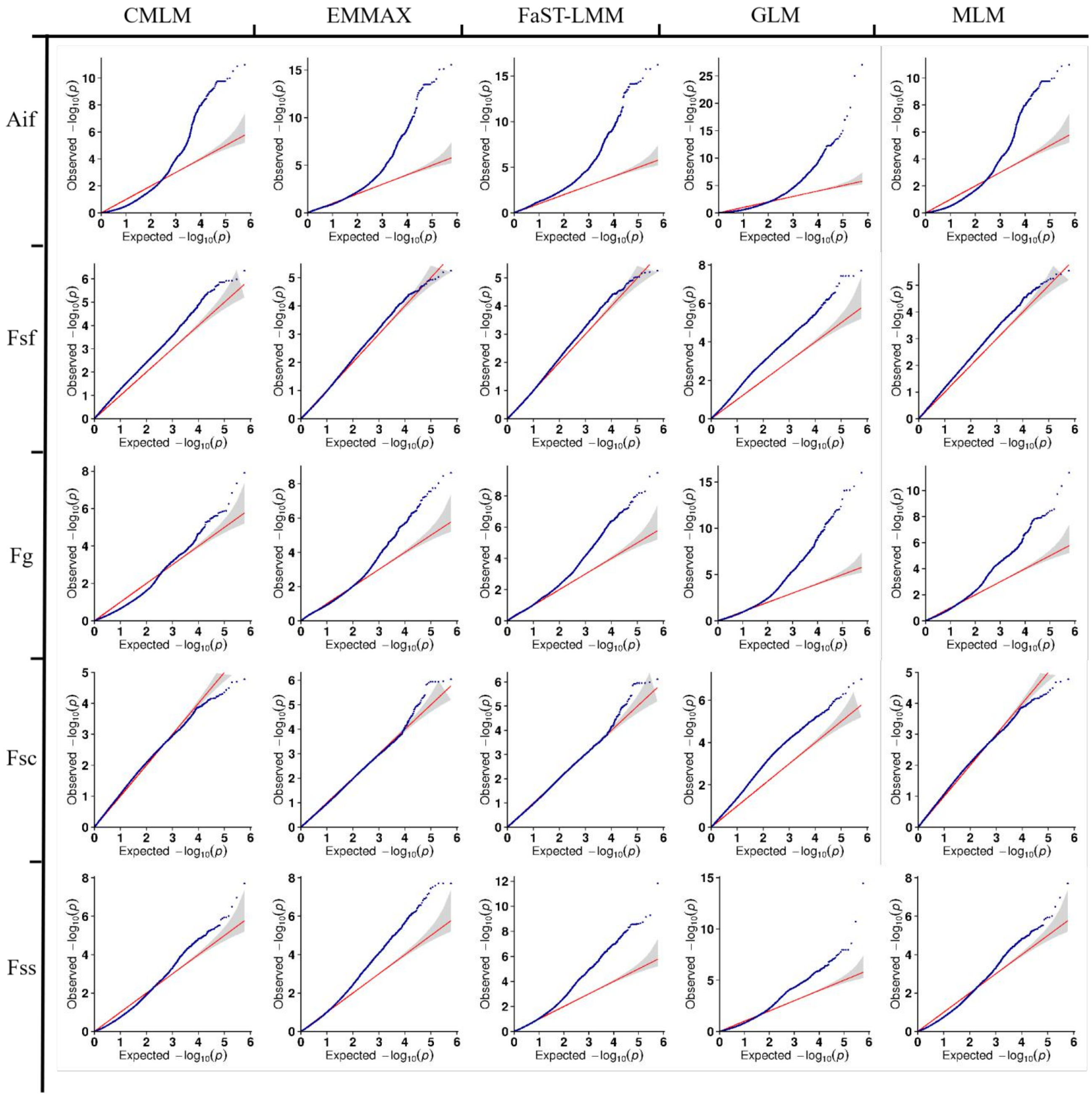

Supplementary Figure 14: Q-Q plots of fruit-related agronomic traits (a). Aif, anthocyanin on immature fruit; Fsf, fruit surface furrow; Fg, fruit glossy; Fsc, fruit surface characteristics; Fss, fruit shoulder shape.

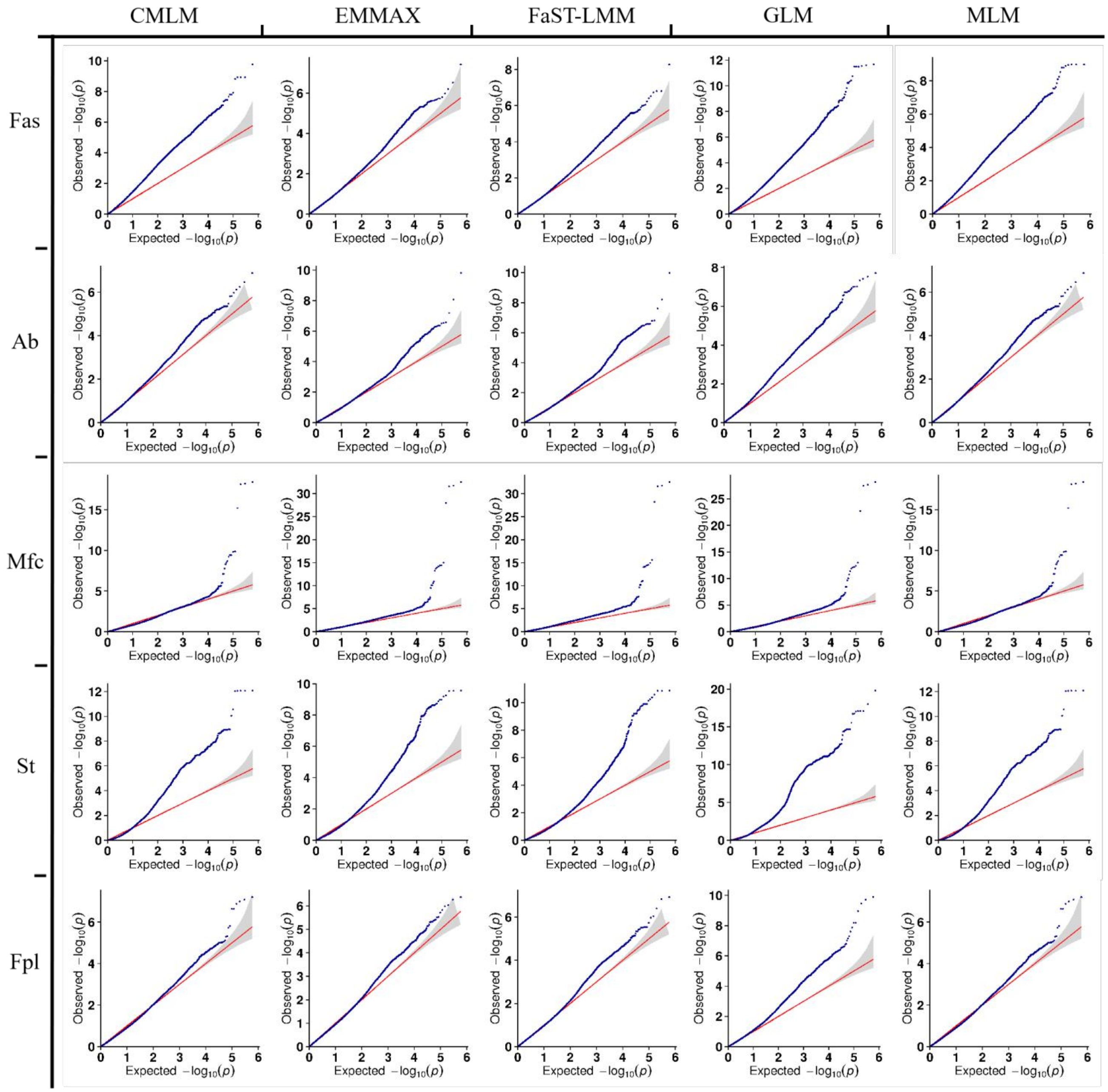

Supplementary Figure 14: Q-Q plots of fruit-related agronomic traits (b). Fas, fruit apex shape; Ab, appendage at blossom-end; Mfc, mature fruit color; St, spicy type; Fpl, fruit pedicel length.

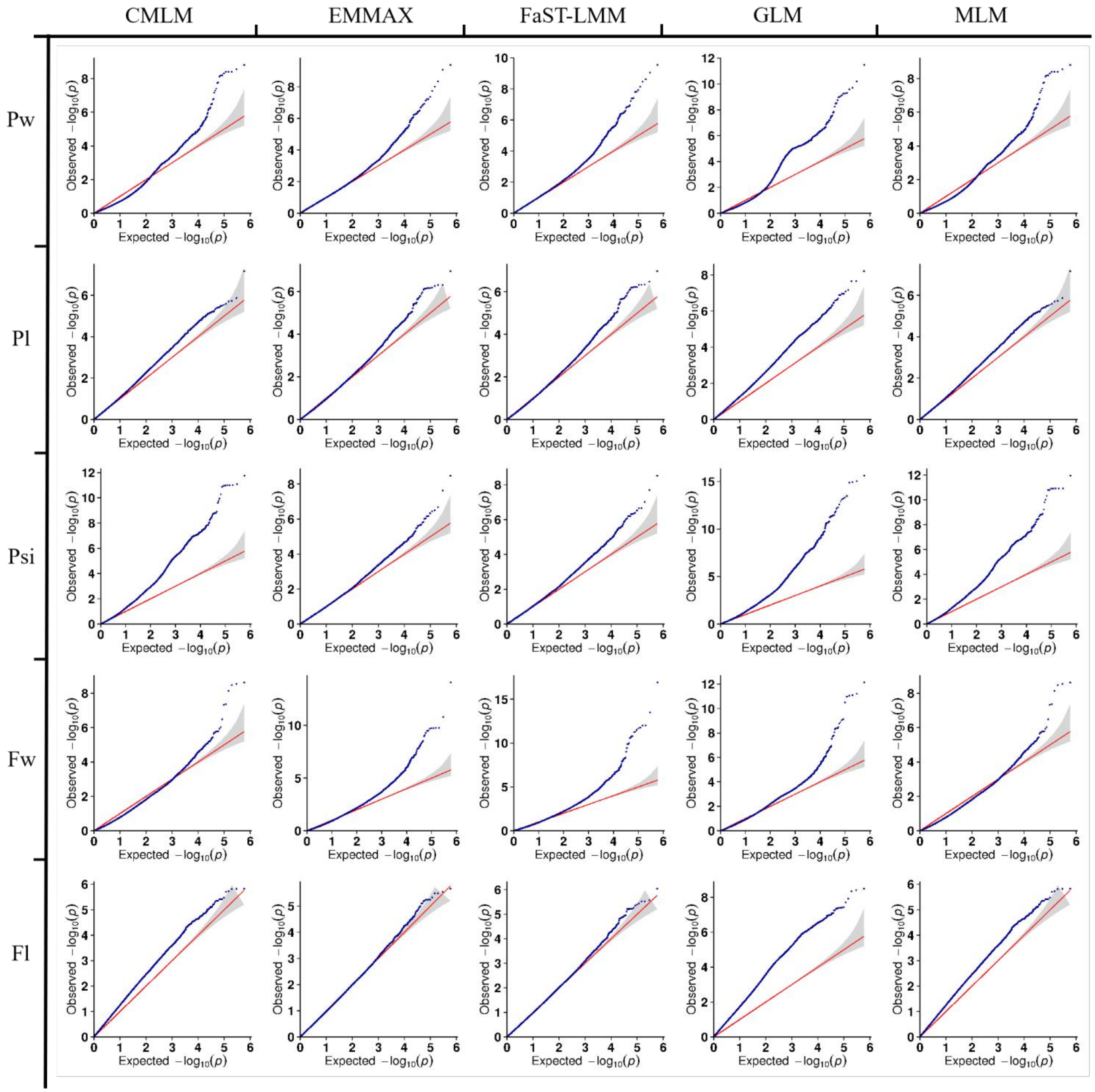

Supplementary Figure 14: Q-Q plots of fruit-related agronomic traits (c). Pw, placenta width; Pl, placenta length; Psi, placenta size index; Fw, Fruit width; Fl, fruit length.

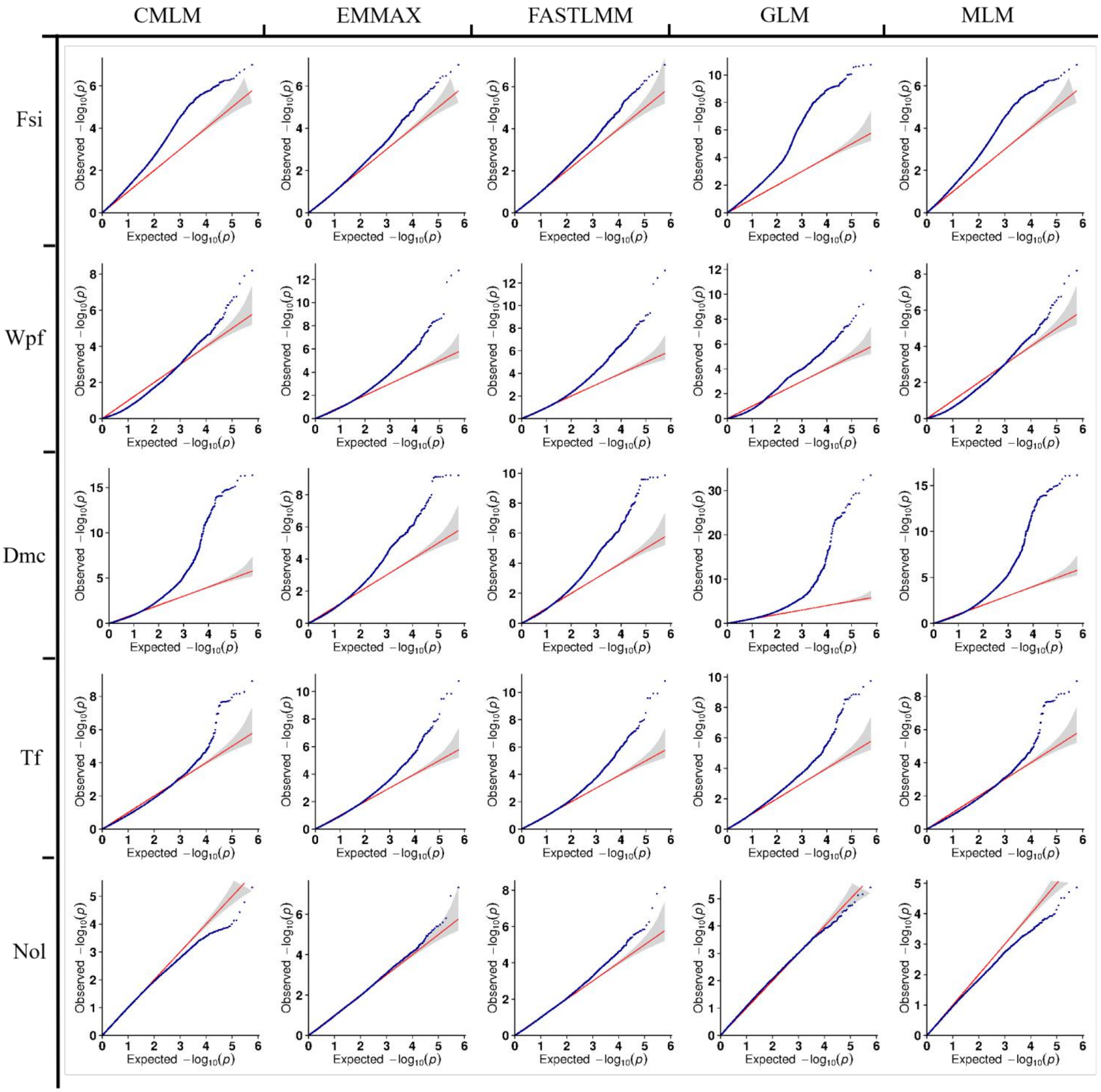

Supplementary Figure 14: Q-Q plots of fruit-related agronomic traits (d). Fsi, fruit shape index; Wpf, weight per fruit; Dmc, dry matter content; Tf, thickness of flesh; Nol, Numeber of locules.

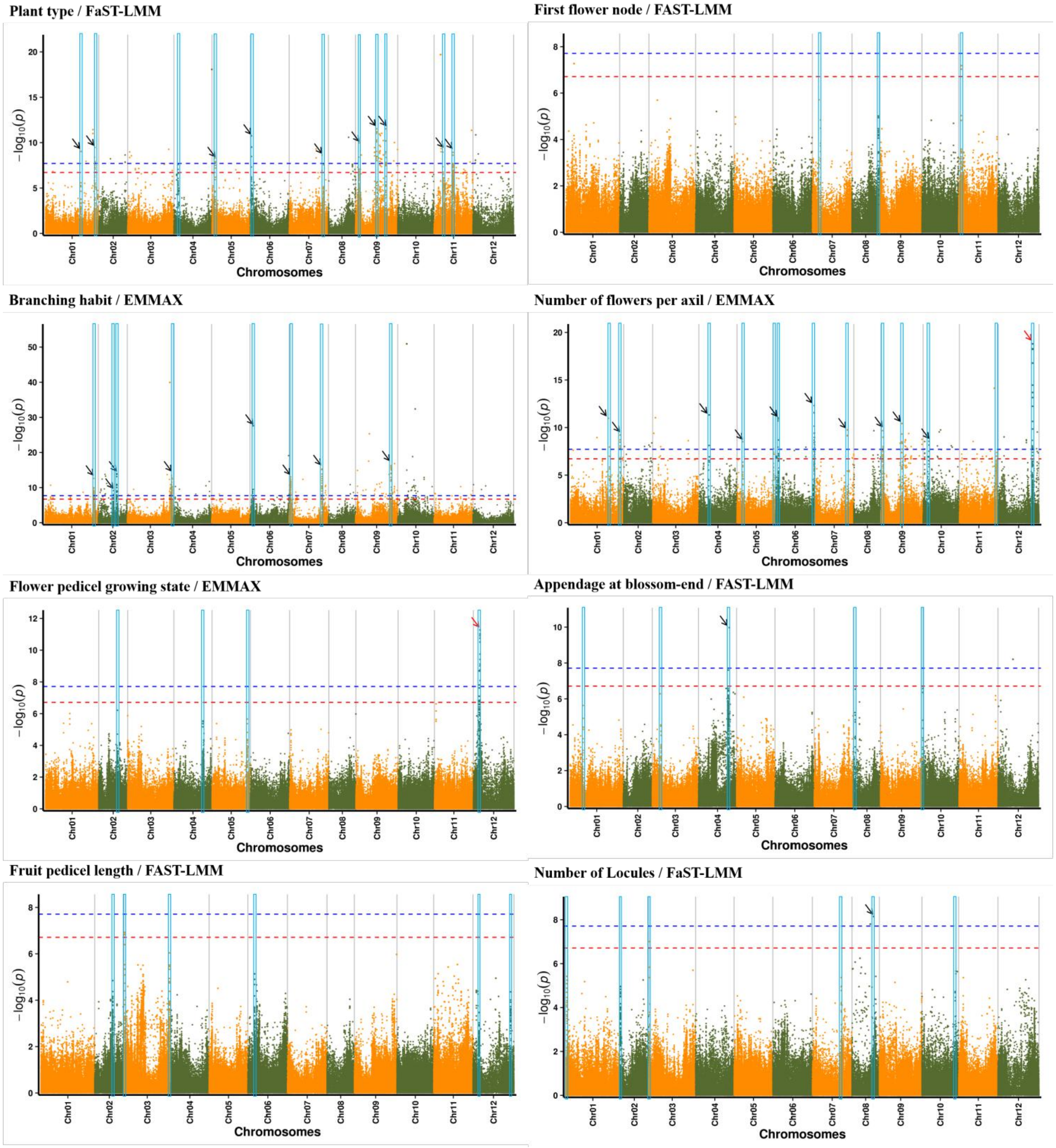

Supplementary Figure 15: Genome-wide association study (GWAS) of plant architecture-related traits by using the optimal statistical model. The highly significant threshold is shown as a dash blue line ($P<1.707*10^{-8}$) and the significant threshold is shown as a dash red line ($P<1.707*10^{-7}$). The blue bar represents associated peak. The black arrow represents a significant associated peak. The red arrow represents the major associated peak.

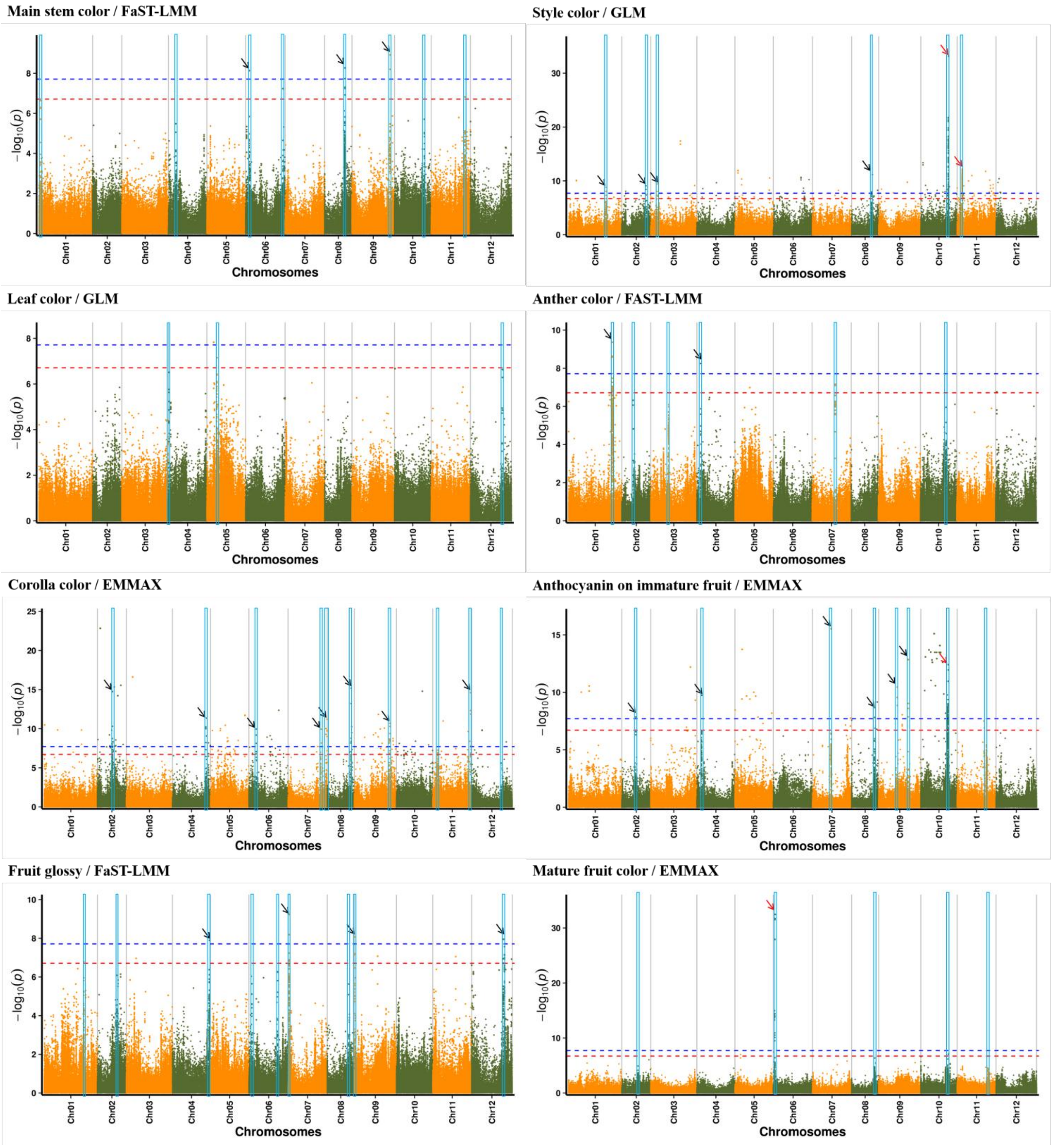

Supplementary Figure 16: Genome-wide association study (GWAS) of color-related traits by using the optimal statistical model. The highly significant threshold is shown as a dash blue line ($P<1.707*10^{-8}$) and the significant threshold is shown as a dash red line ($P<1.707*10^{-7}$). The blue bar represents associated peak. The black arrow represents a significant associated peak. The red arrow represents the major associated peak.

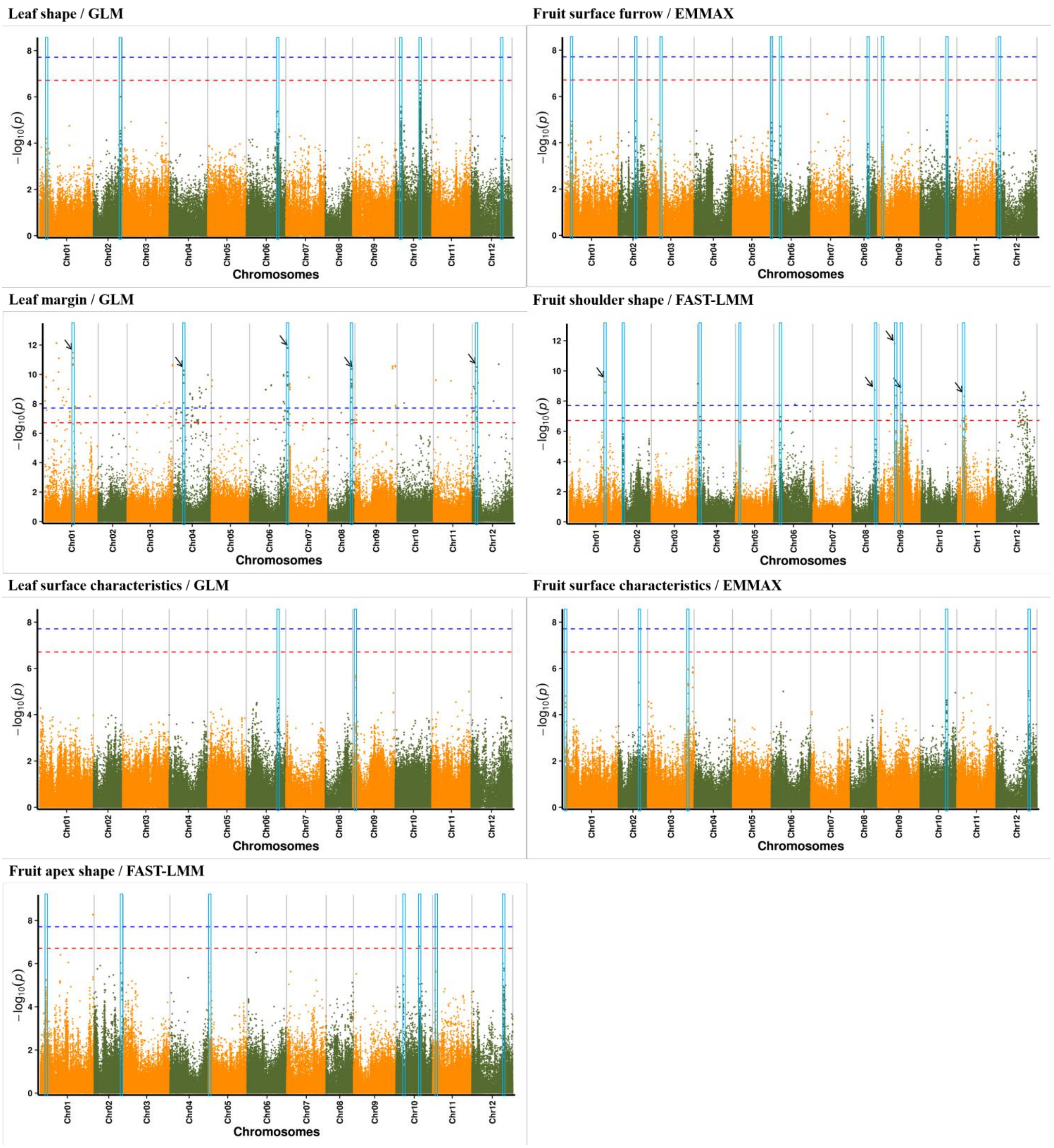

Supplementary Figure 17: Genome-wide association study (GWAS) of shape-related traits by using the optimal statistical model. The highly significant threshold is shown as a dash blue line ($P<1.707*10^{-8}$) and the significant threshold is shown as a dash red line ($P<1.707*10^{-7}$). The blue bar represents associated peak. The black arrow represents a significant associated peak. The red arrow represents the major associated peak.

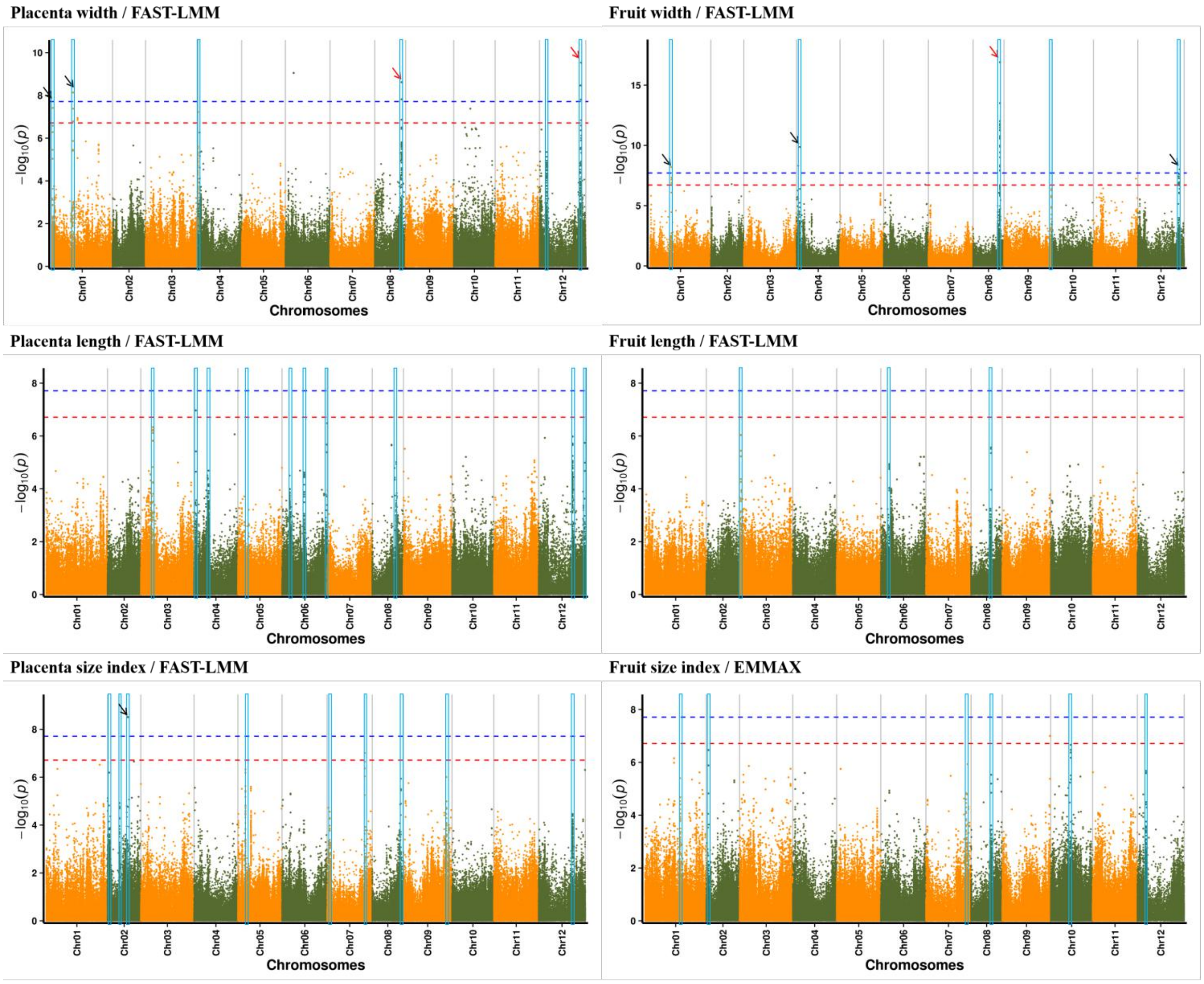

Supplementary Figure 18: Genome-wide association study (GWAS) of size-related traits by using the optimal statistical model. The highly significant threshold is shown as a dash blue line ($P<1.707*10^{-8}$) and the significant threshold is shown as a dash red line ($P<1.707*10^{-7}$). The blue bar represents associated peak. The black arrow represents a significant associated peak. The red arrow represents the major associated peak.

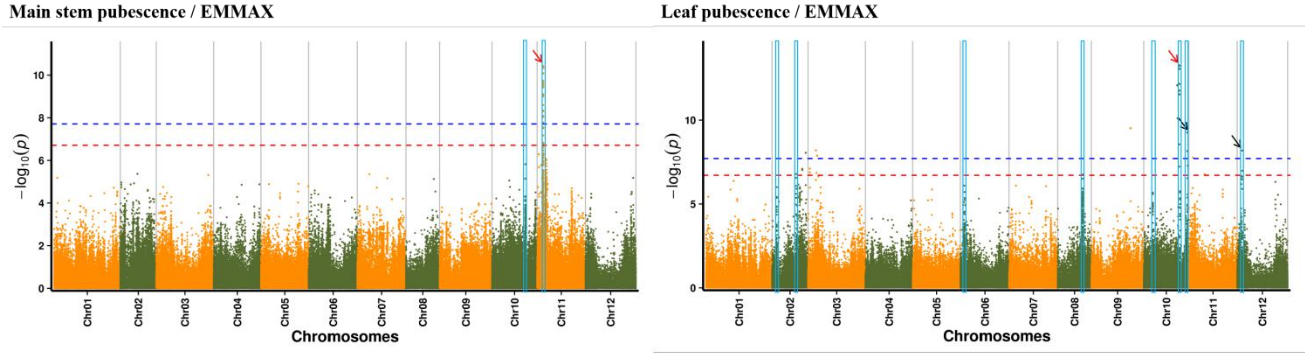

Supplementary Figure 19: Genome-wide association study (GWAS) of pubescence-related traits by using the optimal statistical model. The highly significant threshold is shown as a dash blue line (P<1.707*10^{-8}) and the significant threshold is shown as a dash red line (P<1.707*10^{-7}). The blue bar represents associated peak. The black arrow represents a significant associated peak. The red arrow represents the major associated peak.

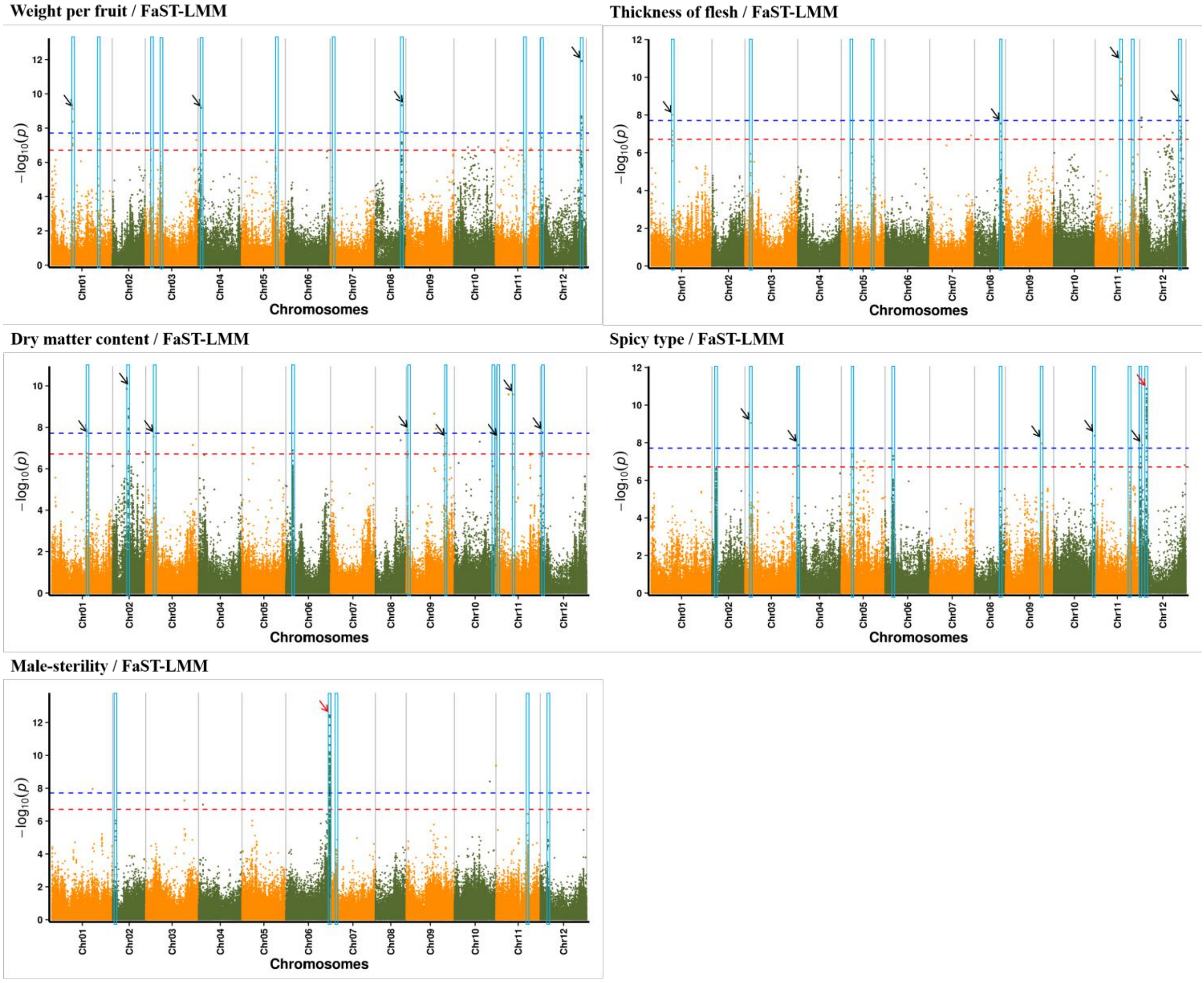

Supplementary Figure 20: Genome-wide association study (GWAS) of others category traits by using the optimal statistical model. The highly significant threshold is shown as a dash blue line ($P<1.707*10^{-8}$) and the significant threshold is shown as a dash red line ($P<1.707*10^{-7}$). The blue bar represents associated peak. The black arrow represents a significant associated peak. The red arrow represents the major associated peak.

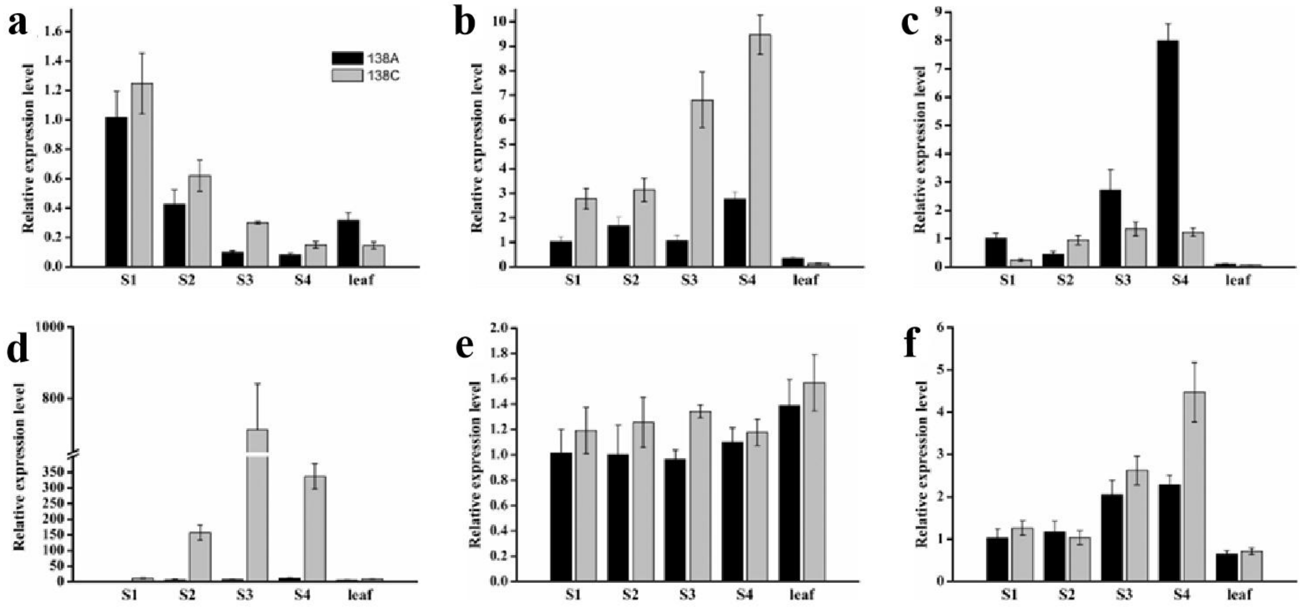

Supplementary Figure 21: Gene expression in leafs and anthers of 138A and 138C at different development stages. S1-S4: represent the tetrad stage, uninucleate stage, binucleate stage and maturing stage in sequence; a: expression analysis of *Capana06g002965*; b: expression analysis of *Capana06g002967*; c: expression analysis of *Capana06g002968*; d: expression analysis of *Capana06g002969*; e: expression analysis of *Capana06g002970*; f: expression analysis of *Capana06g002972*.

1.3 Supplementary Gif

Supplementary Gif 1: Principal component analysis of 287 pepper accessions.**(Shown in Gif)**

1.4 Supplementary Tables

Supplementary Table 1: Current status of gene mapping for agronomic traits in pepper.(**shown in excel)**

Supplementary Table 2: The list and phenotypic data of 287 pepper accessions sampled in this study.(**shown in excel)**

Supplementary Table 3: Descriptive statistic analysis for 23 qualitative traits in 287 pepper accessions.

Plant Organ	Traits	Code	CV	H`	K	K`	PCP/%
Stem	Plant type	Pt	0.09	0.17	3	3	100

	Branching habit	Bh	0.09	0.15	2	2	100
	Main stem color	Msc	0.32	1.07	6	6	100
	Main stem pubescence	Msp	0.54	1.03	4	4	100
Leaf	Leaf shape	Ls	0.29	0.94	3	3	100
	Leaf color	Lc	0.15	0.60	4	5	80
	Leaf margin	Lm	0.32	0.38	3	3	100
	Leaf pubescence	Lp	0.37	0.46	3	4	75
	Leaf surface characteristics	Lsc	0.32	0.92	3	3	100
Flower	Corolla color	Cc	0.21	0.14	3	3	100
	Style color	Sc	0.43	0.23	2	3	67
	Anther color	Ac	0.24	0.38	2	6	33
	Flower pedicel growing state	Fpgs	0.33	0.98	3	3	100
	Male-sterility	Ms	-	-	-	-	-
Fruit	Anthocyanin on immature fruit	Aif	0.38	0.29	2	2	100
	Fruit surface furrow	Fsf	0.40	1.24	4	4	100
	Fruit glossy	Fg	0.11	0.18	2	2	100
	Fruit surface characteristics	Fsc	0.38	1.07	3	3	100
	Fruit shoulder shape	Fss	0.74	0.86	4	5	80

Fruit apex shape	Fas	0.21	0.55	3	4	75
Appendage at blossom-end	Ab	0.29	0.36	2	2	100
Mature fruit color	Mfc	0.23	1.10	5	6	83
Spicy type	St	0.26	0.65	2	2	100

Supplementary Table 4: Descriptive statistic analysis for 13 quantitative traits in 287 pepper accessions.

Trait	Code	Mode	Mean	Min	Max	Range	CV	*H*`
First flower node	Ffn	11	12.61	4.00	25.00	21	0.30	1.98
Number of flowers per axil	Nfpa	1	1.25	1.00	7.00	6	0.66	0.45
Fruit pedicel length(cm)	Fpl	—	4.42	1.87	9.59	7.72	0.26	2.00
Placenta width(cm)	Pw	—	1.39	0.26	5.73	5.47	0.65	1.87
Placenta length(cm)	Pl	—	3.76	0.47	18.18	17.71	0.83	1.57
Placenta size index	Psi	—	0.16	0.01	0.95	0.95	0.96	1.60
Fruit width(cm)	Fw	—	3.45	0.74	10.07	9.33	0.71	1.80
Fruit length(cm)	Fl	—	12.03	1.37	27.55	26.19	0.52	2.01
Fruit shape index	Fsi	—	5.36	0.52	19.82	19.31	0.75	1.90
Weight per fruit(g)	Wpf	—	47.37	0.71	240.20	239.49	1.07	1.58
Dry matter content(%)	Dmc	—	7.71	1.16	39.18	38.02	0.58	1.75
Thickness of flesh(mm)	Tf	—	2.67	0.57	8.99	8.43	0.52	1.93

Number of locules	Nol	3	2.98	2	5	3	0.27	1.17

Supplementary Table 5: Sequencing data statistics and sequencing quality assessment of 287 pepper accessions.(**shown in excel)**

Supplementary Table 6: Data statistics of SLAF tags for each pepper accession.(**shown in excel)**

Supplementary Table 7: Data statistics of SNPs for each pepper accession.(**shown in excel)**

Supplementary Table 8: Grading for all accessions based on SLAF tag number, SNP number, SNP integrity and SNP heterozygosity ratio.(**shown in excel)**

Supplementary Table 9: Diversity evaluation of 13 subgroups.(**shown in excel)**

Supplementary Table 10: Candidate genes and locus information for 36 agronomic traits.(**shown in excel)**

Supplementary Table 11: Distribution of SNPs significantly associated with 36 agronomic traits.(**shown in excel)**

Supplementary Table 12: Annotation of important candidate genes for 36 agronomic traits.(**shown in excel)**

Supplementary Table 13: Annotation information about the eight candidate genes for Ms (*Rf*).

Gene ID	Gene location	Gene annotation
Capana06g002963	214,928,883-214,932,088	alpha/beta-Hydrolases superfamily protein
Capana06g002965	215,102,194-215,102,754	N/A
Capana06g002967	215,172,352-215,173,332	CW-type Zinc Finger
Capana06g002968	215,328,827-215,334,970	Tetratricopeptide repeat (TPR)-like superfamily protein
Capana06g002969	215,340,394-215,341,013	N/A
Capana06g002970	215,676,676-215,694,157	CLIP-associated protein

Capana06g002971	215,710,457-215,719,703	phytochrome interacting factor 3-like 5
Capana06g002972	215,720,523-215,725,395	HIT-type Zinc finger family protein

Supplementary Table 14: Value of correlation in pairwise traits for 36 traits in pepper.(**shown in excel)**

Supplementary Table 15: Detail information of pleiotropic candidate genes in different traits.(**shown in excel)**

Supplementary Table 16: Details of polymerization region for 23 agronomic traits in pepper.(**shown in excel)**

SupplementaryTable 17: Number of polymerization regions for pairwise traits.(**shown in excel)**

Supplementary Table 18: Comparison of the gene mapping results of 36 agronomic traits with those in previous study.(**shown in excel)**

Supplementary Table 19: Assignment standard of 23 qualitative traits in 287 pepper accessions.

Traits	Phenotypic type and assignment criteria
Plant type	1= Spreading; 2= Semi-erect; 3= Erect;
Branching habit	1= Determinate; 2= Indeterminate;
Main stem color	1= Yelloish green; 2= Light green; 3= Green; 4= Dark green; 5= Green with purple stripe; 6= purple;
Stem pubescence	1= None; 2= Sparse; 3= Intermediate; 4= Dense;
Leaf shape	1= Obvate; 2= Long obvate; 3= Lanceolate;
Leaf color	1= Light green; 2= Green; 3= Dark green; 4= Green with purple;
Leaf margin	1= Entire; 2= Undulate; 3=Serrate;
Leaf pubescence	1= None; 2= Sparse; 3= Dense;

Leaf surface characteristics	1= Smooth; 2= Slightly wrinkling; 3= Wrinkling;
Corolla color	1= White; 2= Light green; 3= Purple;
Style color	1= White; 2= Blue; 3= Purple;
Anther color	1= White; 2= Yellow;
Flower Pedicel growing state	1= Pendant; 2= Horizontal; 3= Erect;
Male-sterility	1= Absent; 2= Present;
Anthocyanin on immature fruit	1= Absent; 2= Present; 3= Purple; 4= Purplish black;
Fruit surface furrow	1= None; 2= Shallow; 3= Intermediate; 4= Deep;
Fruit glossy	1= Absent; 2= Present;
Fruit surface characteristics	1= Smooth; 2= Slightly wrinkling; 3= Wrinkling;
Fruit shoulder shape	1= None; 2= Nearly Flat; 3= Sunken; 4= Deep Sunken;
Fruit apex shape	1= Pointed; 2= Blunt; 3= Sunken;
Appendage at blossom-end	1= Absent; 2= Present;
Mature fruit color	1= Orange; 2= Tangerine; 3 = Bright red; 4= Dark red； 5= Chocolate yellow;
Spicy type	1= Pungent; 2= Sweet;

Supplementary Table 20: Primers used in this study.(**shown in excel)**
