# Supplementary figures and images for: Genome-Wide Correlation of 36 Agronomic Traits in the 287 Pepper (Capsicum) Accessions Obtained from the SLAF-seq-Based GWAS
Source: Int J Mol Sci. 2019 Nov 13;20(22):5675. doi: 10.3390/ijms20225675 (PMC6888518; doi:10.3390/ijms20225675)

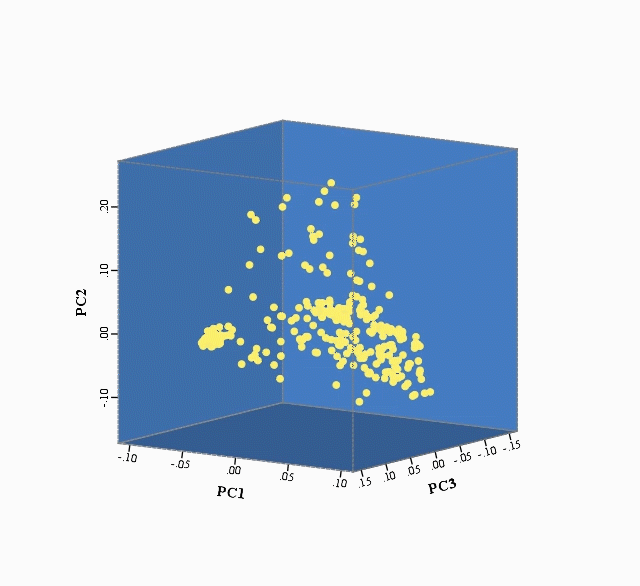

Supplement: Supplementary file 1 [file ijms-20-05675-s001.zip › ijms-611185-proofreading-si/supplementary material/Supplementary gif 1 PCA analysis.gif]
